# Compartmentalized Homeostasis Drives High Bamboo Forest Productivity under Nutrient Imbalance

**DOI:** 10.1002/advs.202517442

**Published:** 2025-12-12

**Authors:** Zhikang Wang, Quan Li, Man Shi, Marcio F.A. Leite, Xinli Chen, Eiko E. Kuramae, Viviane Cordovez, Tingting Cao, Chenglei Zhu, Libin Zhou, Wenjuan Yu, Zhiyao Tang, Changhui Peng, Xinzhang Song

**Affiliations:** ^1^ State Key Laboratory for Development and Utilization of Forest Food Resources Zhejiang A&F University Hangzhou 311300 China; ^2^ Swammerdam Institute for Life Sciences (SILS) University of Amsterdam Amsterdam 1012 WP The Netherlands; ^3^ Department of Microbial Ecology Netherlands Institute of Ecology (NIOO‐KNAW) Wageningen 6708 PB The Netherlands; ^4^ State Key Laboratory of Lake and Watershed Science for Water Security, Nanjing Institute of Geography and Limnology Chinese Academy of Sciences Nanjing 211135 China; ^5^ State Key Laboratory for Vegetation Structure, Function and Construction (VegLab), Institute of Ecology, College of Urban and Environmental Sciences Peking University Beijing 100871 China; ^6^ Department of Biology Sciences, Institute of Environment Sciences University of Quebec at Montreal Montreal Quebec H2×3J8 Canada

**Keywords:** forest productivity, nutrient limitation, Phyllostachys edulis, plant‐microbe interactions, stoichiometric homeostasis

## Abstract

Stoichiometric homeostasis, the ability to maintain internal nutrient balance, is central to plant fitness under soil nutrient variability. While traditionally viewed as static, emerging theory posits that it is a conditionally flexible trait, though empirical evidence is scarce. Through large‐scale field investigations, nutrient additions, and data synthesis, this study shows that Moso bamboo (*Phyllostachys edulis*), a fast‐growing plant species, employs a unique compartmentalized homeostasis strategy by decoupling nitrogen (N) and phosphorus (P) regulation across tissues. It achieves strict N:P homeostasis in leaves while allowing P flexibility in woody tissues to serve as reservoirs that buffer leaves from soil P limitation and microbial competition. This mechanism, consistently observed in bamboo across wide geographical and soil nutrient gradients, yields lower leaf N:P variability than 75 out of 91 co‐occurring tree species, can be one of the critical factors for sustaining ≈25% higher annual productivity than other forests (including evergreen‐broadleaf, deciduous‐broadleaf, and coniferous forests). These findings reconcile classical views of stoichiometric homeostasis and plasticity by demonstrating a flexible, compartmentalized mechanism that resolves growth‐stability conflicts. Recognizing such flexible strategy advances the understanding of eco‐evolutionary feedbacks in ecosystem stoichiometry and improves predictions of species adaptability, nutrient cycling, and carbon sequestration under global change.

## Graphical abstract

This study reveals how Moso bamboo achieves high productivity despite nutrient‐poor soils. It employs a unique strategy, maintaining strict nutrient balance in its leaves while using woody tissues as flexible storage reservoirs. This compartmentalized mechanism buffers the plant from soil phosphorus limitation and microbial competition, resolving the classic conflict between rapid growth and internal stability.

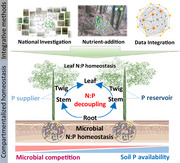

## 1 Introduction

Stoichiometric homeostasis (*H*) is a fundamental ecological concept that describes the ability of an organism to regulate internal elemental concentrations and ratios so as to maintain internal nutrient and metabolic stability amid varying environmental resource availability ^[^ ^1^, ^2^, ^3^ ^]^ This homeostatic mechanism is essential for sustaining vital functions in terrestrial ecosystems, such as stability, ^[^ ^4^ ^]^ productivity, ^[^ ^5^ ^]^ nutrient cycling, ^[^ ^6^ ^]^ and carbon (C) storage. ^[^ ^7^ ^]^ However, environmental fluctuations, such as nitrogen (N) deposition, phosphorus (P) limitation, and climate change, significantly alter nutrient balances, thereby affecting plant *H* ^[^ ^8^, ^9^, ^10^ ^]^ and causing cascading effects on ecological processes from cellular to ecosystem scales.^[^ ^4^, ^9^ ^]^ Therefore, understanding the formation of *H* and its underlying drivers under nutrient imbalance is important to predict ecosystem stability and productivity.

**Figure 1:**
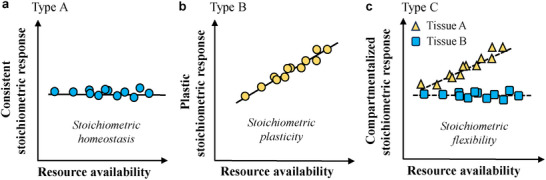
Ecological hypotheses on stoichiometric homeostasis versus plasticity in shaping the stoichiometric responses of organisms to resource availability. a) Species type A (horizontal blue dots) maintains consistent internal elemental ratios regardless of environmental fluctuations, reflecting strict homeostasis but slower growth under nutrient‐limited conditions, as seen in some heterotrophs and K‐strategists. ^[^ ^1^, ^12^ ^]^ b) Species type B (slanted yellow dots) adjusts internal nutrient ratios to match external conditions, demonstrating stoichiometric plasticity for opportunistic growth under nutrient‐rich conditions, typical of autotrophs and *r*‐strategists. c) Species type C exhibits compartmentalized responses to changes in resource availability, showing tissue‐specific flexibility (e.g., tissue A exhibits plasticity while tissue B shows homeostasis). Lines represent species‐specific linear regressions of internal nutrient status against resource availability. The lower slope of the fitting line, the higher homeostasis of the species.

Traditional frameworks, rooted in ecological stoichiometry (**Figure** **1**), posit that plants can exhibit strict homeostasis (type A: maintaining consistent internal ratios regardless of fluctuations, typical of slow‐growing K‐strategists with constrained growth under nutrient limitations) or stoichiometric plasticity (type B: adjusting ratios to match external conditions for opportunistic growth, typical of fast‐growing *r*‐strategists). ^[^ ^1^, ^9^, ^11^, ^12^, ^13^ ^]^ Within this view, plant *H* is considered as a fixed trait, constrained externally by soil nutrient availability and internally by physiological limits, thus homeostasis and plasticity are viewed as mutually exclusive. However, many species display both stability and flexibility depending on tissue type, life stage, or environmental conditions, ^[^ ^2^, ^14^, ^15^, ^16^ ^]^ suggesting unresolved flexibility in nutrient regulation.

The closest attempt to explain this unresolved flexibility comes from emerging eco‐evolutionary theories, ^[^ ^17^, ^18^ ^]^ which propose that *H* can shift over short timescales in response to both genetic variation and environmental drivers. This “stoichiometric flexibility” allows plants to adjust balances across different elements ^[^ ^19^, ^20^ ^]^ while maintaining function, scaling from individuals to ecosystems and influencing growth responses to perturbations. ^[^ ^7^, ^21^ ^]^ Whereas traditional views and the broad concept of flexibility often consider the organism as a single unit, such flexibility is also reflected in flexible regulation across plant compartments. ^[^ ^22^, ^23^ ^]^ Yet, a clear mechanistic framework capturing this context‐dependent regulation is lacking, and empirical support remains limited, primarily due to challenges in designing experiments that control for genetic variation while encompassing a wide range of environmental conditions for a given species. ^[^ ^7^, ^24^, ^25^ ^]^ This gap is particularly evident in fast‐growing species, where unresolved questions persist about how these mechanisms enable the balance between rapid growth and stoichiometric stability, as highlighted by the growth rate hypothesis linking high growth to specific elemental demands. ^[^ ^26^ ^]^

To bridge this gap and provide a mechanistic framework, we introduce the concept of compartmentalized homeostasis (Figure 1c). This concept moves beyond the general observation of stoichiometric flexibility ^[^ ^7^, ^21^ ^]^ by defining a specific regulatory strategy, namely the decoupling of elemental regulation both by tissue and element, enabling an organism to simultaneously maintain strict homeostasis for critical functions in one compartment while exhibiting plasticity in another to serve as a buffer against nutrient imbalance. It allows for adaptive flexibility without sacrificing metabolic stability, thereby resolving the perceived trade‐off between growth (associated with plasticity) and stability (associated with homeostasis) that is central to the Type A/B paradigm. However, this strategy is context‐dependent, co‐shaped by internal adjustments (genetic variation, cross‐tissue allocations, allele‐specific expression) and external drivers (environmental variations, soil nutrients, microbial interactions), aligning with the theory of rapid evolution in stoichiometric phenotypes. ^[^ ^17^, ^18^ ^]^

If compartmentalized homeostasis effectively works on the growth‐stability tradeoff, we posit that it should be a key functional trait underlying high and stable productivity in some fast‐growing species under nutrient limitations. Such a mechanism would provide a competitive advantage by enabling efficient nutrient use and sustained growth under imbalanced conditions, potentially explaining the ecological success and wide adaptation of certain species. Testing this link between internal nutrient regulation strategies and ecosystem‐level productivity is therefore critical for advancing predictive ecology. Moso bamboo (*Phyllostachys edulis*) offers an ideal model to apply this concept. It is a fast‐growing clonal species thriving in N‐rich but P‐limited conditions, showing general adaptations and low genetic diversity across subtropical regions. ^[^ ^27^, ^28^ ^]^ It thus offers a unique model system to test compartmentalized homeostasis at a large continental scale with highly heterogenous environments while controlling for genetic variation. Moreover, Moso bamboo achieves rapid growth (finish height growth up to 20 meters within 2 months) and higher annual productivity (up to 6.03 t·ha^−1^·yr^−1^) than other subtropical tree species, ^[^ ^29^, ^30^ ^]^ despite of wide P limitation. We therefore hypothesize that bamboo exploits this flexible strategy to resolve the growth–stability trade‐off, enabling high productivity under nutrient‐imbalanced conditions.

To test our hypothesis, we disentangle internal and external effects using integrated approaches ^[^ ^31^ ^]^ with Moso bamboo forest as a model system. For the first time, we i) conducted nationwide field investigations across 27 Moso bamboo populations, covering forest area over 4 million hectares, to identify plant‐soil‐microbe stoichiometry patterns and test if plants adopt compartmentalized homeostasis across heterogenous environments; ii) validated our findings via a nutrient‐addition experiments simulating N and P gradients while controlling the population and environment ^[^ ^27^ ^]^; and iii) integrated literature data from other forests to compare the nutrient regulation strategy and subsequent productivity differences. We define stoichiometric response as homeostatic if the nutrient concentration in the bamboo plant tissues is not affected by the nutrient availability in soil. However, the stoichiometric response is considered plastic if the nutrient concentration in the different bamboo tissue is significantly affected by the soil nutrient availability.

## 2 Results

### 2.1 Patterns of the Overall C: N: P Stoichiometry in Plant, Soil, and Microorganisms

**Figure 2:**
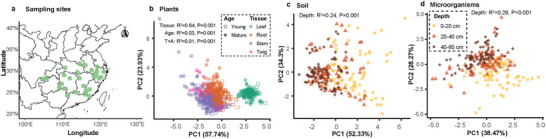
Sampling sites of Moso bamboo representative populations a) and principal coordinates analysis of variations in stoichiometric traits (C, N, and P concentrations and their ratios) in plants b), soil c), and microorganisms d). A total of 81 forest plots were investigated. The sample sizes for plant, soil, and microorganisms are *n* = 648, *n* = 243, and *n* = 243, respectively. The data includes the factors of plant age (young: one‐year‐old bamboo; mature: three‐year‐old bamboo), tissue (leaf, twig, stem, root), and soil depth (0–20 cm, 20–40 cm, 40–60 cm). Statistical significances are tested by PERMANOVA (permutational multivariate analysis of variance) at *P* < 0.05.

Across 27 independent sampling sites in subtropical China (**Figure** **2**a), plant stoichiometric traits (C, N, and P concentrations and their ratios) displayed a strong tissue‐dependent pattern (Figure 2b), particularly between the leaf and non‐leaf tissues (R^2^ = 0.64, *P* = 0.001, permutational multivariate analysis of variance [PERMANOVA]). Distance‐to‐centroid analysis on PCoA scores showed that leaf samples (mean distance = 0.60) were substantially less dispersed than other tissues (1.00, 1.07 and 1.30 for twig, root and stem, respectively) (*P* < 0.05, by permutation multivariate dispersion test). Plant N and P were more concentrated in the leaves than in other tissues, with young bamboo exhibiting significantly higher concentrations than mature bamboo (Figures S1 and S2, Supporting Information, *p* < 0.05). Soil and microbial stoichiometry exhibited clear depth‐dependent trends (R^2^ = 0.24‐0.26, *p* = 0.001): the variability in soil and microbial samples decreased with increasing soil depth (Figure 2c,d) and soil total C, N, and P concentrations and microbial biomass C (MBC), N (MBN), and P (MBP) generally decreased with depth (Figure S3, Supporting Information). Empirical semi‐variograms revealed distinct spatial scales of variability for stoichiometric traits in soil, plants, and microbial communities (Figure S4, Supporting Information). Soil displayed spatial structure across broader distances and also substantial microscale heterogeneity. Plant semi‐variance increased with inter‐site distance and generally stabilized at the largest sampling separations, while microbial biomass indicated small‐scale patchiness and limited spatial dependence.

### 2.2 Stoichiometric Homeostasis (*H*) of Phosphorus and Nitrogen in Plants and Microorganisms under Heterogenous Nutrient Levels

**Figure 3:**
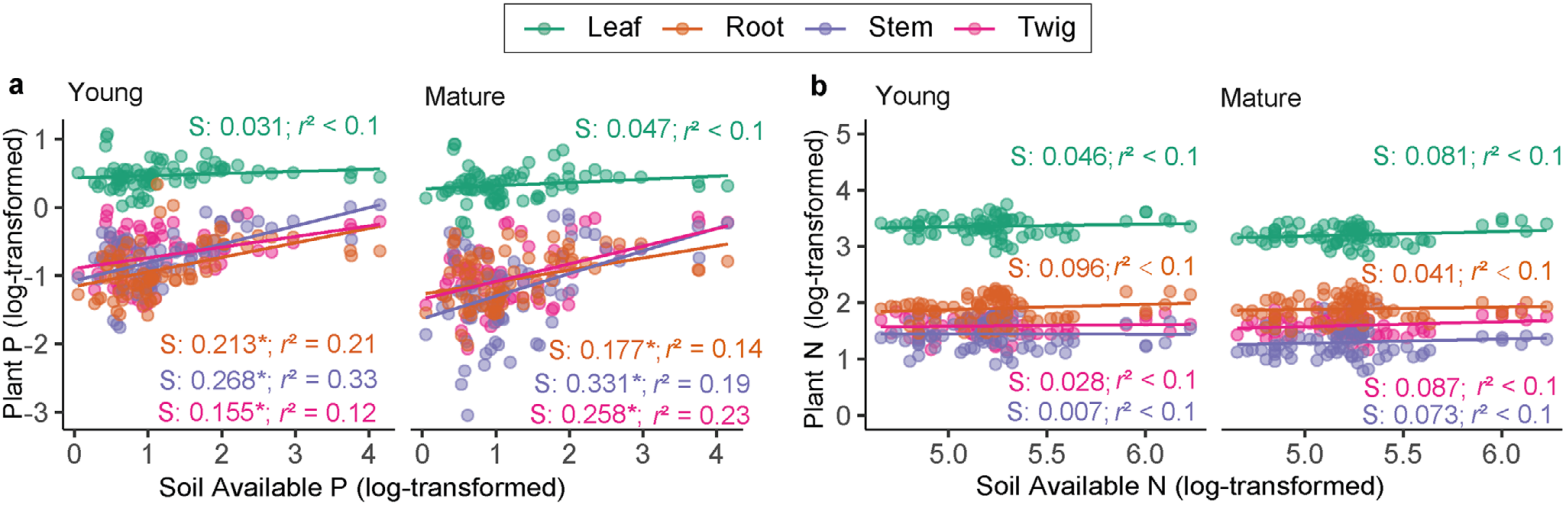
Plant P a, *H_plantP_*) and N b, *H_plantN_*) homeostasis in response to changes in soil available P and N, respectively. Plant P and N in different tissues (leaf, root, stem, and twig) for young (one year old) and mature (three years old) bamboo were compared upon soil available N and P at top soil layer (0–20 cm). The median values of soil available P and available N were 2.69 mg g^−1^ (range from 0.37–63.3 mg g^−1^) and 185.65 (range from 38.8–507.1 mg g^−1^) at top soil across all sites, respectively. The slopes (*S*, i.e., model coefficients) of the linear regression models indicate the *H_plantP(N)_* of different tissues (*n* = 81 for each regression), and the asterisks (*) indicate significance at *p* < 0.05. The data were transformed based on natural logarithms. The detailed information of the linear regression models is listed in Table S1 (Supporting Information). The results of *H_plantP_* and *H_plantN_* in deeper soil (20–40, and 40–60 cm) remain consistent (Figure S5, Supporting Information).

Different plant compartments showed distinct stoichiometric responses in their P and N concentrations as a result of changes in soil available P and N (**Figure** **3**). *H_plantP_* (plant P homeostasis, defined as the slope of linear regression between plant P and soil available P) remained strictly homeostatic in leaves (0.02 < *S* < 0.07) but was more sensitive in other tissues (0.14 < *S* < 0.33) (Figure 3a), whereas *H_plantN_* for all tissues consistently exhibited strict homeostasis (0.001 < *S* < 0.13, Figure 3b). Such patterns were consistent across different soil depth or plant age (Figures S5 and S6, Supporting Information). Most of the sites showed low soil available P (SAP_median_ = 2.7 mg kg^−1^, SAP_mean_ = 5.5 mg kg^−1^), except for sites YY and ZG, with soil available P concentrations of 49.5 and 15.4 mg kg^−1^, respectively, and our analysis showed that the root, twig, and stem responded to the changes in P.

For microorganisms, *H_micN_* (microbial N homeostasis, defined as the slope of the linear regression between microbial biomass N and soil total N) exhibited higher homeostasis (*H_micN_* < 0.25) than *H_micP_* (*H_micP_* < 0.5); however, *H_micP_* was more sensitive to changes in soil depth and increased from 0.178 at 0–20 cm to 0.376 at 40–60 cm (Figure S7, Supporting Information). According to the microbial homeostasis framework, ^[^ ^32^ ^]^ this could be attributed to most microbes being under P‐limited and N‐sufficient conditions in the surface layer (0–20 cm), whereas P limitation could be alleviated with increased soil depth (Figure S8, Supporting Information). Interestingly, internal N:P homeostasis was observed in both plants and microorganisms at almost all sites; however, the sensitivity of microorganisms was site‐dependent (Figure S9, Supporting Information).

### 2.3 Tissue‐Specific Homeostasis Is a Strategy to Cope with Microbial Homeostasis

**Figure 4:**
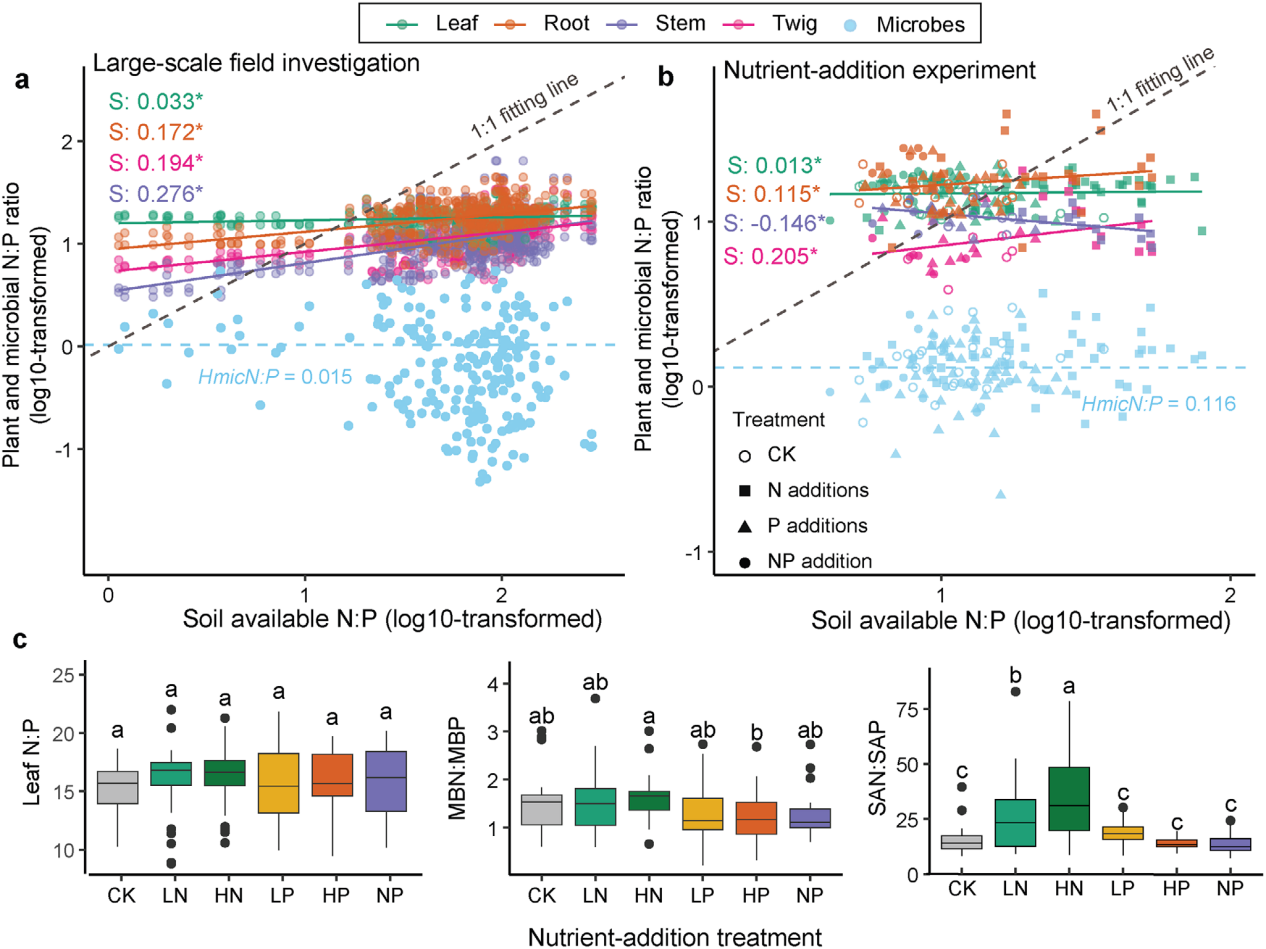
Plant and microbial N:P ratios in response to soil available N:P changes in natural heterogeneous forests and nutrient‐addition field experiment. a) Homeostasis analysis of plant and microbes in the national investigation. The plant data are presented by different tissue (leaf, root, stem, and twig, *n* = 162) for all ages and the microbial data are presented by three depths (0–60 cm, *n* = 243). The effects of plant age, sampling site, and soil depth are presented in the supplementary material (Figure S12, Supporting Information), incorporating these factors does not affect the main conclusion, namely leaf and microbes show N:P homeostasis while other tissues exhibit plasticity. b) Tests of homeostasis in plant tissues and microbes by nutrient‐addition experiment. The plant data are presented by different tissues (leaf, root, stem, and twig, n = 342 for all ages), and the microbial data from two depths (0–40 cm, *n* = 216) are presented by different treatments. The blue dashed line represents the mean *H_micN:P_* and the grey dashed line represents the one‐to‐one plant‐soil relationship_._ According to the plant‐microbe interaction framework, ^[^ ^33^ ^]^ those points above the dashed line indicate relative N limitation and those below the dashed line indicate relative P limitation. Note that both axes have a logarithmic scale, and the asterisks (*) indicate significance at *p* < 0.05. () Differential effects of nutrient additions on N:P ratios in plant‐soil‐microbe system. The statistical significance was tested by post‐hoc test at *p* < 0.05. The treatments are: CK (no addition), HN (high N input), HP (high P input), LN (low N input), LP (low P input), and NP (N and P input). SAN: soil available nitrogen; SAP: soil available phosphorus.

**Figure 5:**
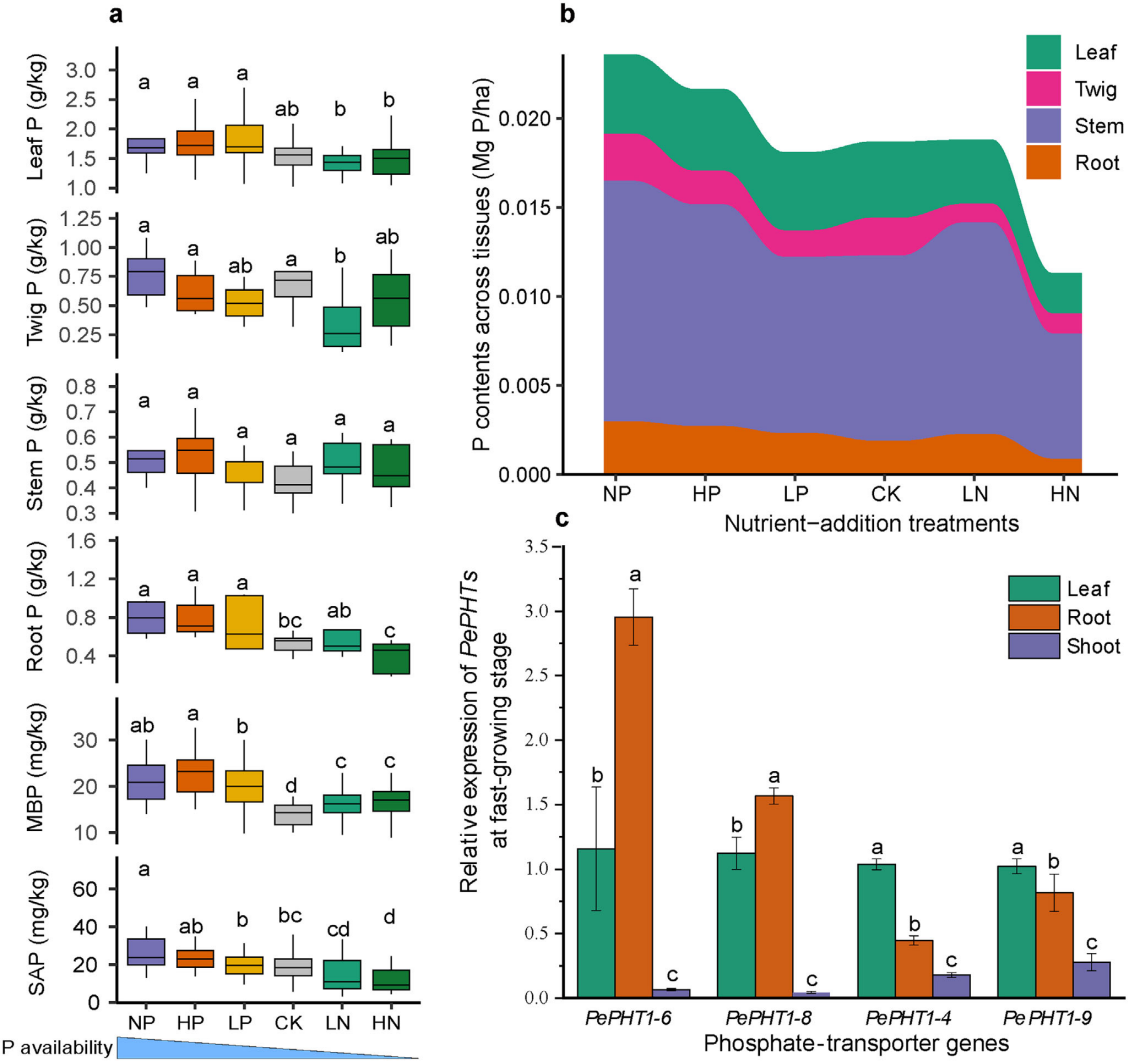
Effects of nutrient additions on P status in plant‐soil‐microbe system. a) Total P concentrations in different plant tissues (*n* = 342) and microbial biomass P (*n* = 216) under different levels of soil available P (SAP) as induced by nutrient‐addition treatments. b) Allocation P contents (Mg P ha^−1^) across different tissues in nutrient‐addition experiment. This was calculated from the P concentration and plant biomass. c) *PePHT*s (phosphate‐transporter genes) relative expression across leaf, root, and shoot tissues (*n* = 12) during the fast‐growing stage. ^[^ ^34^ ^]^ The effects were assessed by ANOVA and the statistical significance was tested by post‐hoc test at *p* < 0.05.

Both *H_plantN:P_* in leaves and *H_micN:P_* in the microbial community consistently exhibited strong homeostasis to the change in soil N:P (**Figure** **4**a), whereas non‐leaf tissues exhibited higher plasticity, suggesting tissue‐specific homeostasis of both P and N:P in plants. Given the homeostasis of microbial community, we expect that the plant‐microbiome associations play an important role in regulating this tissue‐specific homeostasis. To test whether external variations (soil microorganisms and soil nutrient level) influence plant and microbial homeostasis in bamboo ecosystem, we further compared the plant‐microbe relationships and tested their stoichiometric homeostasis in a controlled nutrient‐addition experiment with different amounts of N and P inputs. Regardless of the type of nutrients added to the soil, *H_plantN:P_* in leaves remained at the highest level of homeostasis (*H* < 0.1) and *H_micN:P_* remained stable (Figure 4b). Nutrient addition showed no significant effect on leaf N:P and microbial N:P (*P* > 0.05; Figure 4c), but roots and twigs increased the N:P ratio after N additions (Figure S10, Supporting Information). N‐addition treatments caused no significant change in leaf N or P content, whereas P addition synchronically increased leaf N and P content, MBN, and MBP (**Figure** **5** **;** Figure S11, Supporting Information), thus maintaining the plant and microbial N:P balance under wide P‐limited conditions (Figure 4). Our results thus indicate that tissue‐specific homeostasis in bamboo is a critical strategy for coping with stoichiometrically homeostatic microbial communities.

### 2.4 Reallocation of P Contents across Tissues Contributed to Bamboo Leaf N:P Homeostasis under P Limitation

Bamboo leaves maintained a stable N:P ratio under P limitation (we define P limitation based on the status of soil available P and the plant‐microbe interaction framework, ^[^ ^33^ ^]^ see method section for more details) compared to other tissues. We hypothesized that this stability resulted from strategic allocation of P between leaves and other tissues. So, we analyzed the allocation patterns of P across different tissues using reduced major axis (RMA) analysis (Figure S13 and Table S2, Supporting Information), which provides a robust means of quantifying scaling relationships by accounting for measurement error in both variables and offering biologically meaningful slope estimates. The scaling slopes for the correlations between leaf P and P in stems, twigs, and roots were < 0.7 (Figure S13a–c, Supporting Information; *S* = 0.38–0.65), indicating that P‐change rates were higher between leaves and woody tissues than those within the woody tissues themselves. Conversely, correlations among stems, twigs, and roots, had slopes close to 1 (Figure S13d–f, Supporting Information, *S* = 0.81–1.23), suggesting more consistent P allocation within these tissues. This pattern was more pronounced in mature bamboo, which exhibited a higher P‐change rate (i.e., lower slope) than that in young bamboo. N was more conserved than P in both leaf and non‐leaf tissues (Figure S13g–l, Supporting Information).

To confirm field observations, we conducted a nutrient‐additions experiment to exaggerate P limitation by increasing excessive N input, which generally decreased soil P availability, P concentrations in plant‐microbe system, and absolute P content in bamboo compared to high‐P treatment (Figure 5). This indicates that under extremely contrasting nutrient‐imbalanced conditions (e.g., high P versus high N), N and P concentrations in leaves might be reshaped synchronously (Figure S11, Supporting Information), but still exhibiting N:P homeostasis (Figure 4c). Moreover, N additions increased MBP compared to the control (CK) while showed no significant improvement on plant leaf P (Figure 5a), indicating strong microbial competition for P under P limitation. Conversely, high P additions (treatments HP and NP) increased P contents in non‐leaf tissues (Figure 5b), indicated a stored P pool in woody tissues under P enrichment. P additions increased the bamboo biomass (Figure S14, Supporting Information) at both individual and ecosystem level, thus indicating the alleviative effects on P limitation.

### 2.5 The Roles of N‐ and P‐Transporters in Regulating N‐P Allocation across Tissues

At the fast‐growing stage of bamboo, the expression levels of P‐transporters (*PePHTs*) showed a tissue‐specific pattern that underpins the observed P allocation (Figure 5c). The high expression of *PePHT1‐6* and *PePHT1‐8* in roots suggests a primary role in P uptake from the soil, whereas the expression of *PePHT1‐4* and *PePHT1‐9* in shoots indicates a specific role in P transport and loading into woody tissues, facilitating their function as a P reservoir. Similarly, homologous gene of N‐transporters (*PeNRTs*) showed high expression patterns (e.g., *PeNRT2.4*) in shoots supporting sustained N transport to maintain leaf N:P homeostasis (Figure S11c, Supporting Information).

In response to nutrient additions, P‐transporters (*PePHT1‐4* and *PePHT1‐8*) remained stable in leaves, but were significantly enhanced in twigs under N addition and in roots under P additions (Figure S15a–f, Supporting Information). In contrast, N‐transporters (*PeNRT2.4* and *PeNRT5.18*) were more sensitive to P additions (treatments NP, HP, and LP) while showing stable under N additions (treatments LN and HN), with such patterns observed across different tissues (Figure S15g–i, Supporting Information). This coordinated gene expression provides the molecular basis for the compartmentalized allocation of N and P.

### 2.6 Linking Leaf N:P Homeostasis to Bamboo Ecosystem Productivity with Comparisons to Other Forests

**Figure 6:**
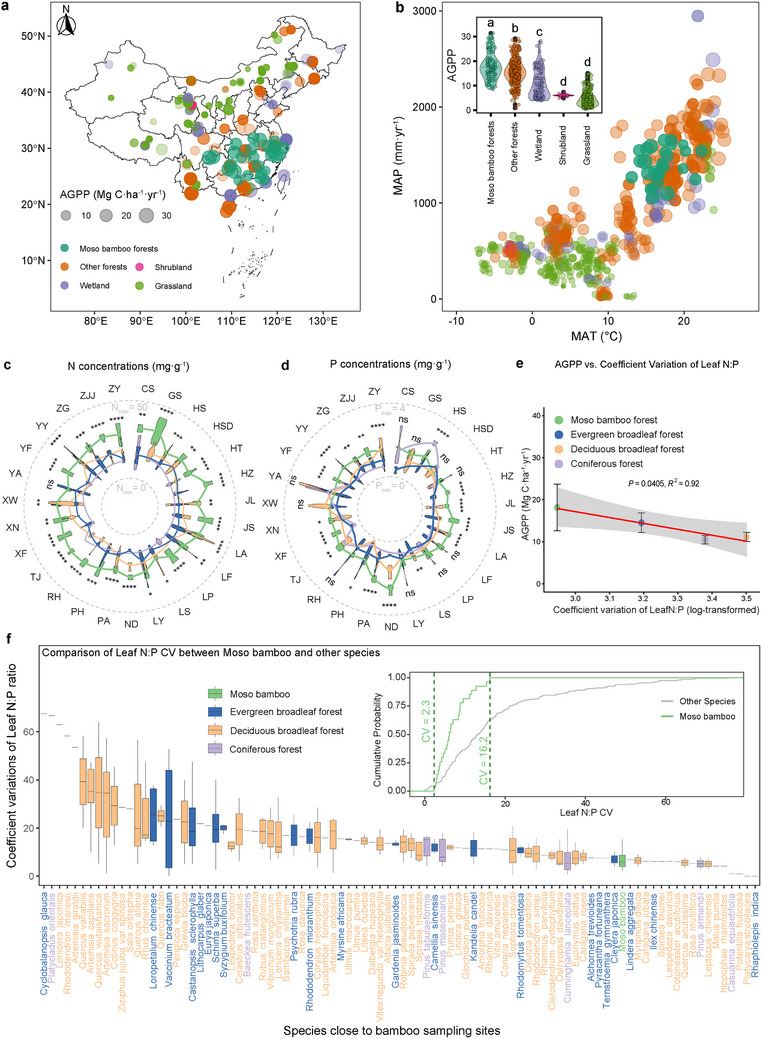
Comparisons between bamboo forests and other terrestrial ecosystems regarding leaf nutrient status, N:P homeostasis, and productivity. a) Annual gross primary productivity (AGPP, Mg·ha^−1^·yr^−1^) mapped in Moso bamboo forests (*n* = 81), other forests (*n* = 292), shrublands (*n* = 13), grasslands (*n* = 291), croplands (*n* = 175), and wetlands (*n* = 101) in China. b) Effects of mean annual temperature (MAT) and mean annual precipitation (MAP) on AGPP in all ecosystems. The data of terrestrial ecosystem AGPP was obtained from published literature. ^[^ ^35^ ^]^ c,d) N and P concentrations (mg·g^−1^) in the leaves of bamboo and other tree species that grow near bamboo populations, including coniferous trees (Conifer, *n* = 51), deciduous broadleaf trees (DeciBr, *n* = 257), and evergreen broadleaf trees (EverBr, *n* = 418). The inner dashed grey rings mean ‘*y* = 0′ for both N and P concentrations, the outlier dashed grey ring means ‘*y* = 50′ for N concentrations and ‘*y* = 4′ for P concentrations, respectively. The data of plant N and P concentrations in other representative subtropical tree species that near to bamboo sampling site (± 1° longitude or altitude) was obtained from previous studies. ^[^ ^36^, ^37^ ^]^ The asterisks (*, **, ***, ****) indicate the significance levels at *p* < 0.05, 0.01, 0.001, and 0.0001, respectively. e) Linking coefficient variations (CV) of ecosystem‐based leaf N:P to AGPP in different forests. The data of ecosystem‐based leaf N:P and AGPP in other ecosystems were extracted from previous studies. ^[^ ^38^, ^39^ ^]^ The data of Bamboo forests’ AGPP was estimated by field investigation in this study (3885 bamboo culms from 81 plots at 27 sites). f) Comparison of CV in leaf N:P between Moso bamboo and other plant species (*n* = 91) in the neighborhood forests (listed in Table S3, Supporting Information). Cumulative probability of Moso bamboo and other species were plotted to predict the possibility of CV in bamboo leaf N:P lower than that in other species.

Moso bamboo forests also demonstrated superior productivity compared to other forests, shrublands, grasslands, and wetlands in China (P < 0.01, one‐way ANOVA) (**Figure** **6**a). We speculated that compartmentalized homeostasis plays a major role in this success, which can be summarized in three hypotheses: (Hi) geographical location and climate traits may drive this high productivity; (Hii) stoichiometric homeostasis in plant leaves is linked to productivity; (Hiii) the monoculture of bamboo supports higher productivity compared to other natural forests.

To test Hi, we compared the annual gross primary productivity (AGPP) between bamboo forests and other terrestrial ecosystems in China by integrating the published data. The results showed that bamboo populations spanned a wide range of MAT and MAP values (Figure 6b), but no positive effects of mean annual temperature (MAT) (effect size = ‐1.03, P = 0.001) and mean annual precipitation (MAP) (effect size = ‐0.004, P = 0.20) were noted on AGPP by linear mixed‐effect models.

To test Hii and Hiii, we compared leaf nutrient status of bamboo and other representative functional group species (*n* = 149) in the neighbor subtropical forests (within ± 1° latitude or longitude of 27 sampling sites). Bamboo leaves exhibited higher N concentrations in 26 sites and higher P concentrations in 17 sites (*p* < 0.05), regardless of whether the neighboring forests were coniferous, deciduous, or evergreen (Figure 6c,d; Figure S16, Supporting Information). In addition, bamboo outperformed both natural and plantation forests in terms of leaf N concentrations and exhibited comparable P concentrations while maintaining a stable N:P ratio (*p* < 0.05; Figure S17, Supporting Information). This N–P balance resulted in significantly lower coefficient variations in bamboo leaf N:P ratios than that in other species (*p* < 0.05, Figure 6f) and likely contributed to the higher AGPP than other forests (R^2^ = 0.92, *p* = 0.04; Figure 6e). The random forest model revealed ecosystem twig P contents as the most critical factor driving AGPP (importance = 13.15%, Figure S18, Supporting Information), confirming our previous results (Figure 5) and suggesting that twig P may serve as a reservoir for maintaining leaf N:P balance and plant growth under P limitation.

Transporting P from twigs to leaves uses existing vascular tissues and specific transporter proteins. This process generally requires less metabolic energy than overcoming the strong binding of P to soil particles and also to compete against the soil microbes (who are already maintaining their stoichiometric homeostasis).

## 3 Discussion

### 3.1 Tissue‐Specific and N‐P Decoupling Mechanisms of Compartmentalized Homeostasis

A central question in forest ecology is how plant tends to be flexible under nutrient imbalance (or limitation). Our results demonstrate that the bamboo model system sustains leaf N:P stability under nutrient imbalance while enabling rapid growth across a wide range of environmental influences. This rapid growth (low‐*H* trait) coexists with strict leaf N:P homeostasis (high‐*H* trait), contradicting Type A/B hypotheses (Figure 1) and lacking a unified explanation for its stoichiometric regulation. We thus propose the compartmentalized homeostasis, a tissue‐dependent concept derived from previously established stoichiometric flexibility hypothesis, ^[^ ^7^ ^]^ enables different homeostasis level of various elements and tissues, with the joint contribution of genetic variation and environmental influences. ^[^ ^17^ ^]^ In this framework, the “conditionality” of nutrient regulation does not imply that the plant switches strategies, but that the expression of homeostasis versus plasticity is differentially allocated across tissues in a context‐dependent manner.

In the case of bamboo, we are able to single out the environmental factors while controlling the genetic variations in the framework of compartmentalized homeostasis. The conditionality in bamboo reflects as a tissue‐specific strategy decoupling stable N regulation across tissues from flexible P reallocation. The N homeostasis was achieved despite variations in soil N or P availability, soil depth, plant tissues, and age group, align with previous theories where nutrient‐poor environments frequently trigger homeostasis for essential elements. ^[^ ^40^ ^]^ At the molecular level, this strict N homeostasis is supported by the high expression of nitrate transporters (e.g., *PeNRT2.4* and *PeNRT7.14*) in shoots (Figure S11c, Supporting Information), which facilitates efficient N translocation to leaves and stable N:P level to uphold photosynthetic function. ^[^ ^41^ ^]^ This molecular capacity is synergistically enhanced by morphological adaptations like the robust root system ^[^ ^42^ ^]^ and clonal integration. ^[^ ^34^ ^]^ In contrast, P shows strict homeostasis in leaves but plasticity in woody tissues, suggesting an adaptive evolution beyond strict homeostasis, ^[^ ^17^ ^]^ to optimize nutrient acquisition across tissues for growth and essential metabolisms under imbalanced N‐P condition, ^[^ ^12^, ^14^ ^]^ aligning with our previous hypothesis on productivity‐nutrient allocation. ^[^ ^39^ ^]^ This N‐P mismatched strategy, evident in subtropical species, ^[^ ^2^ ^]^ suggesting the potential wide adoption of compartmentalized homeostasis and inspiring further works on how phylogenetic variations versus environmental changes in shaping homeostasis across different plants, in addition to the focus on leaf N or P concentrations. ^[^ ^36^ ^]^ It should be noted that, N and P concentrations in leaves might be changed synchronously under extremely contrasting nutrient‐imbalanced conditions, but still exhibiting N:P homeostasis.

Broadening the perspective, this tissue‐specific decoupling aligns with findings from Schreeg et al.,^[^ ^22^ ^]^ where stems, roots, and older leaves exhibited greater N:P responsiveness to soil availability than new foliage, indicating compartmentalized regulation to buffer limitations. Our empirical focus on a fast‐growing species under P limitation extends previously established flexibility hypothesis, ^[^ ^7^ ^]^ showing how resolves growth constraints absent in previous conceptual framework. Consequently, unlike fixed whole‐plant models, ^[^ ^2^, ^9^, ^14^, ^15^, ^16^ ^]^ our data support emerging context‐dependent views, ^[^ ^17^, ^18^ ^]^ yet innovate by demonstrating N‐P decoupling in fast‐growing species, contrasting with stricter homeostasis in slow‐growers. ^[^ ^26^ ^]^ While this strategy proved robust across the wide range of conditions sampled in our study, its efficacy may have limits under extreme co‐limitation by multiple nutrients or abiotic stresses beyond the scope of this work, such as severe drought, which could disrupt the internal transport and reallocation processes that are fundamental to this mechanism. ^[^ ^43^ ^]^

### 3.2 P Reallocation and Competitive P Acquisition over Microbes Support N:P Homeostasis in Bamboo

Internal and external drivers synergistically enable P flexibility and thus compartmentalized homeostasis in bamboo. Nutrient‐addition experiments confirm P storage in stems under P‐enrichment and reallocation under N‐induced P‐limitation. This aligns with other species, such as *Larix gmelinii* var. *principis‐rupprechtii* (larch), by activating alternative P‐acquisition pathways. ^[^ ^44^ ^]^ Moreover, the high P resorption rates (up to 94%) ^[^ ^45^ ^]^ and connections between young and mature bamboo can enhance nutrient reallocation. ^[^ ^42^ ^]^ In this study, young bamboo showed a higher rate of P reallocation than mature bamboo in stem‐leaf and twig‐leaf relationships, consistent with the higher N and P levels observed in young bamboo leaves and their faster growth rates in previous experiments. ^[^ ^29^ ^]^ The distinct expression patterns of phosphate transporters elucidate the mechanism behind this flexible regulation. The high and variable expression of *PePHT1‐6* in roots reflects its role in dynamic P acquisition from the soil. Crucially, the relatively high expression of *PePHT1‐9* in shoots (Figure 5c) and *PePHT1‐4* in twigs (Figure S15, Supporting Information) strongly suggest their specialized roles in P transport and storage within woody tissues, creating the buffer pool that is essential for the compartmentalized homeostasis strategy. Hence, the key internal mechanism underlying this compartmentalized homeostasis is the flexibility of plant for the efficient uptake and reallocation of P across tissues under P limitation.

**Figure 7:**
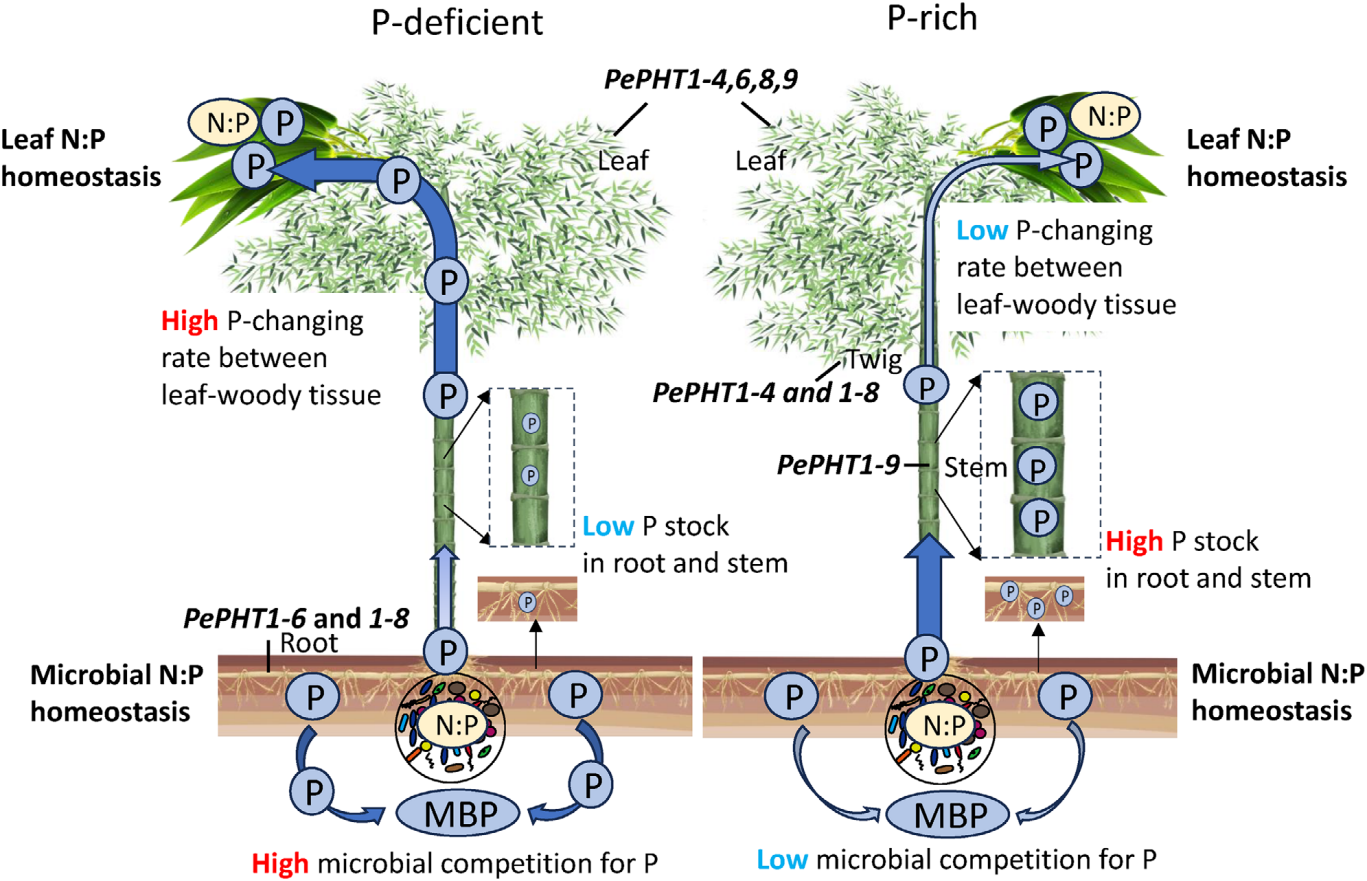
A conceptual diagram for illustrating the mechanisms underlying leaf N:P homeostasis in bamboo forests. Under P deficiency, plant tends to show high changing rate of P between leaf and woody tissues to maintain the leaf P homeostasis, with the help of phosphate transporters (see four main genes *PePHT1‐4, 6, 8, 9*). Soil microorganisms compete for the limited P with plants, leading to lower P stock in the root and stem. Under P‐rich conditions, plant tends to store the excessive P in woody tissues (see gene *PePHT1‐4, 8, 9*) and shows low competition with soil microorganisms. Despite the difference in soil P availability, plant leaves and soil microorganisms exhibit homeostatic N:P ratio under both conditions.

Externally, plants have evolved to establish relationships with other organisms to improve ecological adaptability. ^[^ ^46^, ^47^ ^]^ For instance, soil microorganisms play a role as primary contributors to C–N–P cycling and modulate plant‐soil interactions under different nutrient supplies, correlating with changes in plant *H* and growth performance. ^[^ ^48^, ^49^ ^]^ In bamboo forests, however, their interactions with microorganisms potentially amplify the competition for limited P, as N enrichment intensifies microbial P immobilization (Figure 5a) to sustain their stoichiometric stability. ^[^ ^32^ ^]^ This aligns with a recent study reporting microbial competition for P under elevated carbon dioxide and P‐limited conditions. ^[^ ^50^ ^]^ This external microbial pressure directly reinforces the adaptive value of bamboo's internal strategy: by maintaining a flexible P pool in woody tissues, bamboo can circumvent direct competition with microbes in the rhizosphere and ensure a reliable P supply to leaves, thereby upholding leaf N:P homeostasis despite strong microbial competition (**Figure** **7**). Moreover, bamboo can outcompete microbes in P absorption under N addition by facilitating P cycling through resorption and litter return, as indicated in our previous study. ^[^ ^51^ ^]^ In other P‐limited terrestrial ecosystems, ^[^ ^52^ ^]^ like larch plantations where microbes boost phosphatase activity to alleviate P limitation for plants, ^[^ ^44^ ^]^ or broadleaf invasions where bamboo alters rhizosphere communities for competitive P acquisition. ^[^ ^53^ ^]^ This plant‐microbe interdependence likely supports N:P homeostasis by employing flexible mechanisms ^[^ ^54^, ^55^ ^]^ to selectively assimilate nutrients in exchange for secreted C from plants. In our case, both plant and microorganisms eventually stabilized at the N:P ratio regardless of nutrient addition.

### 3.3 Compartmentalized Homeostasis Supports Competitive Productivity in Bamboo Forests

Species exhibiting stronger stoichiometric homeostasis tend to sustain higher and more stable biomass, with ecosystems dominated by such species often showing greater productivity and stability. ^[^ ^4^, ^14^ ^]^ This is seen in some K‐strategists, ^[^ ^1^, ^9^, ^17^ ^]^ such as *Pinus sylvestris* and *Betula pendula*. ^[^ ^56^ ^]^ Here, compartmentalized homeostasis bridges two paradigms: strict homeostasis and flexibility. ^[^ ^1^, ^12^, ^17^ ^]^ By decoupling N and P regulation across tissues, bamboo exploits compartmentalized homeostasis and achieves higher productivity and stability than most neighboring tree species (Figure 6a,f). This is synergistically enhanced by unique physiological traits: a robust root system that provides structural and storage capacity, ^[^ ^42^ ^]^ efficient clonal integration that enables nutrient translocation between ramets, ^[^ ^34^ ^]^ and high nutrient resorption rates. ^[^ ^45^ ^]^ These traits, which are not commonly seen in other co‐occurring trees, collectively allow lower variation in bamboo leaf N:P, optimized nutrient‐use efficiency, and reduced metabolic costs, supporting dominance in heterogeneous environments ^[^ ^2^, ^39^ ^]^ and enhanced biomass production. ^[^ ^12^ ^]^

This offers a mechanistic resolution to an apparent paradox posed by the Growth Rate Hypothesis (GRH). The GRH posits that rapid growth is linked to high P demand for ribosomal RNA synthesis, ^[^ ^26^ ^]^ which typically necessitates high P uptake and can lead to stoichiometric plasticity under P limitation. Moso bamboo, however, maintains both rapid growth and strict leaf N:P homeostasis under widespread low soil available P. We demonstrate that this is achieved not by defying the GRH, but by evolving a compartmentalized P regulation strategy that satisfies the high P demand of growth. By storing and flexibly reallocating P from woody tissues to leaves, bamboo effectively creates an internal P reservoir that buffers the ribosomal RNA machinery in meristems and leaves from external P scarcity. This mechanism ensures that the P supply for growth is maintained (supporting the GRH) while simultaneously stabilizing the leaf N:P ratio critical for photosynthesis and metabolic stability. The flexible responses may be a widespread adaptation for those fast‐growing plants in ecosystems where spatial or temporal nutrient heterogeneity favors adaptive flexibility, ^[^ ^17^ ^]^ as maintaining balance in one nutrient (e.g., N) while being flexible in others (e.g., P) ensures both resilience and adaptability. ^[^ ^7^ ^]^ However, modeling ecosystem productivity is complex owing to multiple factors, such as climate, nutrients and genetic variation, ^[^ ^50^, ^57^ ^]^ and predicting productivity with a sole factor, such as leaf N:P, remains challenging. ^[^ ^58^ ^]^ For example, the dominant trees in tropical, subtropical, and temperate regions remain relatively stable leaf N:P ratio while differing in nutrient status and productivity (Table S4, Supporting Information). Consequently, our findings suggested that intraspecific variations in leaf N:P across various environments may be a crucial functional trait associated with plant growth and adaptability.

## 4 Conclusion

Our nationwide investigations followed by nutrient‐addition experiments present strong evidence of compartmentalized homeostasis in Moso bamboo. This mechanism, which decouples different nutrient regulation across tissues while incorporating microbial roles, demonstrates how plants can maintain leaf stoichiometric stability through flexible internal storage, which buffers leaves from soil P limitation and microbial competition for P. It provides a new perspective on traditional stoichiometric homeostasis and plasticity hypotheses with potential implications for predicting ecosystem productivity under nutrient imbalances. Together, the compartmentalized homeostasis in bamboo forests, independent of geographical and soil variations, strengthens our understanding of ecosystem stoichiometric responses in nutrient‐imbalanced ecosystems with potential links to growth‐stability balance under global change.

## 5 Experimental Section

### Large‐Scale Field Investigations and Sample Collections

A total of 81 bamboo forest plots in 27 representative sites were investigated at the national scale (Table S5, Supporting Information), covering 11 provinces and representing an area of over 4 million hectares. Because *H* may vary across plant tissues and ages, and ecosystem productivity cannot be solely predicted by leaf stoichiometric traits, ^[^ ^15^, ^22^, ^58^, ^59^ ^]^ an integrated approach that incorporated geographical variation, plant tissue, age, soil microorganisms, and soil nutrient availability at different soil depths was adopted.

Samples were collected from sites within a longitude of 100–121°E and latitude of 20–35°N. The MAT was 12.5–22.3 °C and MAP was 1027.5–2000 mm. The soil organic C was 4.1–79 mg g^−1^, soil total N was 0.5–6.4 mg g^−1^, available N was 38.8–507.1 mg g^−1^, soil total P was 0.1–1.1 mg g^−1^, and available P was 0.37–63.3 mg g^−1^, with an average pH of 4.88. Soil was sampled at three different depths (0–20, 20–40, and 40–60 cm) using a drill from October to November 2021, with three replicates at each site. The diameter at breast height (DBH) of 3885 independent Moso bamboo culms was measured at all sites. The C, N, and P concentrations were analyzed in 236 soil samples (three depths (0–20, 20–40, 40–60 cm) × three replicates) and 648 plant samples (two ages (1 and 3 years) × four organs (root, stem, leaf, twig) × three replicates). Stones, plant residue, and other debris in each sample (≈500 g) were removed prior to subsequent analyses. Plant roots, stems, twigs, and leaves were harvested separately from three plots at each site for both young (1 year) and mature (3 years) bamboo. All the samples were immediately transferred to the laboratory in a cooling box containing ice bags. The plant samples were then oven‐dried at 65 °C to a constant weight prior to C, N, and P estimations.

### Nutrient‐Addition Experiments

The *H* variation in nature may not reflect variation in the degree of homeostasis ^[^ ^24^ ^]^; therefore, controlled conditions were used to validate the hypothesis by conducting nutrient addition experiments with N and P fertilizers independently or simultaneously to simulate significant gradients of soil N and P levels while maintaining the same population. Here, in December 2021, eighteen “20 m × 20 m” plots were established in the bamboo forests. The forest stand conditions and soil environmental factors were consistent across plots, with a 20 m buffer zone between adjacent plots to minimize interference. Six N and P addition treatments were implemented using ammonium nitrate (NH_4_NO_3_) as the N source and monosodium phosphate (NaH_2_PO_4_) as the P source: control (0 kg P·ha^−1^ year^−1^, CK), low N (LN, 30 kg N·ha^−1^ year^−1^), high N (HN, 60 kg N·ha^−1^ year^−1^), low P (LP, 50 kg P·ha^−1^ year^−1^), high P (HP, 100 kg P·ha^−1^ year^−1^), and a combined N and P treatment (60 kg N·ha^−1^ year^−1^ and 100 kg P·ha^−1^ year^−1^). Each treatment was replicated thrice. The N and P solutions were sprayed once a month starting in January 2022, whereas the control group received an equal volume of water to mitigate the effects of soil moisture variation on the experiment. Plants (roots, stems, twigs, and leaves) and soil samples (0–20 cm and 20–40 cm) were collected in July, 2022, November, 2022, February, 2023, May, 2023, August, 2023, November, 2023, and September, 2025, following the same protocols as in the national field investigation. The plant growth and age information were investigated in October, 2023 and September, 2025. The plant samples at last sampling time point were used for reverse transcription quantitative PCR (qPCR), and the other samples were used for determination of C, N, and P concentrations.

### Determination of C, N, and P Concentrations in Plant, Soil, and Microorganisms

The total C and N concentrations in the plant and soil samples were determined using an elemental analyzer (ElementarVario EL III, Germany). Total P concentrations in the plants and soil were determined using the Kjeldahl method and colorimetrically analyzed at 880 nm after a molybdate reaction. Microbial biomass C (MBC) and N (MBN) were determined using the chloroform fumigation extraction method based on 0.5 M K_2_SO_4_ solution. Microbial biomass P (MBP) was calculated from chloroform‐released inorganic P. Soil available N and P were determined using the alkali N‐proliferation method and molybdenum blue method (Bray P), respectively. ^[^ ^51^ ^]^ Soil moisture content was determined as the mass loss after drying the samples at 105 °C for 24 h. Soil pH was determined in a 1:2.5 (w/v) soil‐to‐water extract using a digital pH meter.

### Homeostasis Coefficients

The plant and microbial homeostasis according to the following formula was calculated (equation (1)):

(1) $$\mathrm{Log}\left(y\right)=\left(\frac{1}{H}\right)\mathrm{Log}\left(x\right)+c$$

where *y* is the plant N or P concentration (% dry mass), microbial biomass N or P, or their N:P ratios. For plant homeostasis calculation, *x* is the available N, available P, or their ratio in the soil; for microbial homeostasis, *x* is the total N, total P, or their ratio in the soil (see supporting information for the results of microbial homeostasis using the soil available N and P); and c is a constant. ^[^ ^1^ ^]^ We compared regression line slopes using the estimated marginal means of linear trends (“emtrends”) function from the R package “emmeans” to test for significant differences in homeostasis between factorial groups. For statistical convenience, slopes (coefficients, *S*) were used to measure the homeostasis level (1/*H*), which can be classified into four types: 0 < *S* < 0.25, steady‐state; 0.25 < *S* < 0.5, weakly steady‐state; 0.5 < *S* < 0.75 weakly sensitive; and *S* > 0.75, sensitive. ^[^ ^8^ ^]^

### Diagnosis of Relative Nutrient Limitation in Plant‐Soil‐Microbe System

Relative nutrient limitation of plant and microbes in bamboo forests was assessed using an integrated approach. Primarily, we evaluated the status of soil available P (SAP) at top layer (0–20 cm) in all bamboo forests (SAP_median_ = 2.7 mg kg^−1^, SAP_mean_ = 5.5 mg kg^−1^) in the context of global terrestrial ecosystems and well‐established P‐limited forests in EucFACE project (9.34  mg kg^−1^). ^[^ ^50^ ^]^ Second, the plant‐microbe interaction framework was applied, ^[^ ^33^ ^]^ which identifies relative nutrient limitation by comparing the N:P ratios of plant leaves (we added the data of twigs, stems, and roots in our framework) and soil microbial biomass against the soil available N:P ratio. In this framework, data points falling below the 1:1 line (plant N:P  = soil available N:P) indicate that plants are more P‐limited than the soil microbial community. This diagnosis was cross‐validated with direct measurements of soil available P concentrations and the growth responses observed in the nutrient‐addition experiment (Figure S14, Supporting Information). We acknowledge that stoichiometric ratios alone can be ambiguous, ^[^ ^60^ ^]^ therefore, this determination of P limitation was based on the consistent evidence from all these approaches.

### Nutrient Allocation Patterns Across Tissues

The N and P contents (i.e., density, Mg·ha^−1^) in various tissues were estimated based on biomass proportions, followed by the method described by Shi et al.,^[^ ^42^ ^]^ To investigate nutrient allocation patterns, the scaling approach was used, Y = a × X^b^. ^[^ ^39^ ^]^ After log transformation, the power function was expressed as a linear regression equation (*ln*Y = a + b × *ln*X). The RMA technique was employed to examine the correlations between log‐transformed nutrient contents, where a slope (b, b_RMA_) <1 indicated that the rate of change in N or P in Y was slower than that in X. Relative N or P content was calculated based on its proportion in the entire plant. ^[^ ^39^ ^]^ This approach was particularly advantageous in stoichiometric studies because it avoids bias introduced by ordinary least squares regression, captures proportional changes between traits, and allows reliable inference of coordination among elemental pools across tissues.

### Reverse Transcription Quantitative PCR (qPCR)

To reveal the molecular mechanisms underlying nutrient transportation across tissues, the plant samples at last sampling time point from nutrient‐addition experiment and the samples from different plant tissues during the fast‐growing stage were collected, as nutrient‐transportation rate has been proved to be high at this stage. ^[^ ^34^ ^]^ For the samples of three bamboo tissues (leaf, root, shoot), total RNA was extracted using a Total RNA Kit (Tianmo, TR205‐50, China). The first strand of cDNA was synthesized using a PrimeScript RT Reagent Kit (Takara, RR037A, Japan). Four phosphate transport genes (*PePHT1*‐4, *PePHT1*‐6, *PePHT1*‐8, and *PePHT1*‐9) and four nitrogen transport genes (*PeNFP2.4*, *PeNFP5.18*, *PeNFP6.4*, and *PeNFP7.14*) were selected for further qPCR analyses (Table S6, Supporting Information). qPCR was performed using the Roche Light Cycler 480 SYBR Green I Master Kit (Roche, 04887352001, Germany) with the specific primers listed in Table S6 (Supporting Information), *PeGAPDH* were used as the internal controls. ^[^ ^61^ ^]^ Three independent experiments were performed. The relative abundance of each gene was calculated from the 2^−ΔΔCT^ values between the target gene and the reference gene. ^[^ ^62^ ^]^

### Estimation of Aboveground Biomass and Annual Gross Primary Productivity

The aboveground individual biomass of Moso bamboo was calculated using a model (Equation (2)) developed from biomass data with a correlation coefficient (*R* ^2^  =  0.937) and accuracy (96.43%, calculated by mean percent standard error and confidence intervals) with 95% confidence intervals. ^[^ ^63^ ^]^

(2) $$f\;\left(D,A\right)=747.787D^{2.771}\;{\left(\frac{0.1484A}{0.028+A}\right)}^{5.555}+3.772$$

where *f* is the aboveground biomass of Moso bamboo, including the leaf, twig, stem, and underground trunk, and *D* and *A* are the DBH (cm) and age of the Moso bamboo, respectively.

The total aboveground biomass per unit area was estimated by multiplying the average individual biomass in each plot by stand density. AGPP was estimated by the total C in the aboveground biomass per unit area for both young and mature bamboo, with a normalized proportion of 0.5042. ^[^ ^28^ ^]^ Notably, this slightly underestimated the AGPP in bamboo forests because of older bamboo culms due to poor management.

### Statistical Analyses

All statistical analyses were performed using R software (version 4.3.1, https://cloud.r‐project.org/) with packages including vegan, lmodel2, lme4, randomForest, and gstat. Data were pre‐processed by checking for normality and homogeneity of variances using the Shapiro‐Wilk test and visual inspection of Q‐Q plots. Where necessary to meet the assumptions of parametric tests, data were log‐transformed prior to analysis, as indicated in the respective figure captions. Outliers were evaluated using the interquartile range but were retained in the dataset as they represented valid biological variation. For scaling analyses and homeostasis calculations, nutrient content values were log‐transformed (natural logarithm) to linearize relationships.

Sample sizes (*n*) for each specific analysis are provided in the corresponding figure captions, main text, or methods descriptions. For the national field investigation, the sample sizes were: plant tissues, *n* = 648; soil, *n* = 243; and microorganisms, *n* = 243. All replicates are biological replicates (independent forest plots, individual bamboo culms, or soil cores), and no data points were excluded from the analyses. It was quantified climatic variables (MAT and MAP) for each site using modelled values sourced from the WorldClim Global Climate database (version 2.1; http://www.worldclim.org). To compare AGPP and climate ubiquity between bamboo forests and other terrestrial ecosystems, we extracted AGPP and climate data for forests (*n* = 292, excluding bamboo forests), shrublands (*n* = 13), grasslands (*n* = 291), croplands (*n* = 175), and wetlands (*n* = 101) from a published database with 181 independent sites. ^[^ ^35^ ^]^ To compare the N and P concentrations between bamboo forests and other forests in the neighborhood (± 1° longitude or altitude close to 27 sampling sites in this study), the data for coniferous (*n* = 51), deciduous (*n* = 257), and evergreen forests (*n* = 418) from previous studies with more than 7000 paired N and P data was extracted. ^[^ ^36^ ^]^ To minimize the unique effects of management and clonal traits on bamboo nutrient concentrations (i.e., pure forest), we integrated the data of leaf N, P concentrations, and N:P ratio from natural forests (broadleaved and coniferous) and planted forests (broadleaved and coniferous) in China by incorporating the data from 127 paired sampling sites. ^[^ ^64^ ^]^

It was employed multiple statistical methods with a significance level (alpha) of *p* < 0.05 for all tests. Principal coordinate analysis (PCoA) was applied to test the dissimilarity of plant, soil, and microbial stoichiometric traits (C, N, and P concentrations and their ratios) across plant factors (age and tissue) and soil depth. The significance of the PCoA was tested using PERMANOVA at *p* value < 0.05. It was quantified within‐group multivariate dispersion as the mean Euclidean distance of samples to their group centroid calculated on PCoA scores. Homogeneity of multivariate dispersions was tested with a PERMDISP (permutation multivariate dispersion) test (999 permutations) to compare the differences of dispersion across plant tissues or soil depth. Empirical semi‐variograms were computed from pairwise site distances and variable residuals using the method‐of‐moments estimator. Variogram models (spherical and exponential) were fitted by weighted least squares using the gstat package in R and model selection was based on lowest residual sum of squares and visual fit.

For comparative analyses, Tukey's honestly significant difference (HSD) post‐hoc test was used for pairwise comparisons following ANOVA. A linear mixed‐effects model was used to estimate the influence of plant tissue, age, soil depth, and climate on homeostasis and AGPP, while accounting for random effects (e.g., sampling site), with model selection based on the Akaike Information Criterion (AIC). A plant‐microbe interaction framework ^[^ ^33^ ^]^ was applied to test the consistent N:P responses of plants and microbes to changes in soil N:P, with the data aggregated from all field sampling sites and all nutrient addition treatments. The significance of the site (or treatment) effects on plant, soil, and microorganisms was evaluated using ANOVA, and the best‐fit model was selected with a threshold of *p* < 0.05. Coefficient variations in leaf N:P were calculated using the mean and standard deviation at each sampling site. Cumulative probability analysis was applied to test the possibility of bamboo leaf N:P variation being lower than that of neighboring species at all field sampling sites. A random forest model with 1000 trees and 10‐fold cross‐validation was applied to identify the important predictors of AGPP, with feature importance measured by the percentage increase in mean squared error (%IncMSE). Coefficient of variation (CV) was calculated as standard deviation/mean, and cumulative probability analysis was used to compare distributions of leaf N:P CV between bamboo and other species.

## Conflict of Interest

The authors declare no conflict of interest.

## Supporting information



Supporting Information

## Data Availability

The data that support the findings of this study are openly available in figshare at 10.6084/m9.figshare.28519541, reference number 28519541. A video for this work is accessible on Youtube (https://youtu.be/48mN6QjdC7c) and Bilibili (https://www.bilibili.com) by searching the title.
