# Supplementary material for: Compartmentalized Homeostasis Drives High Bamboo Forest Productivity under Nutrient Imbalance
Source: Adv Sci (Weinh). 2025 Dec 12;13(11):e17442. doi: 10.1002/advs.202517442 (PMC12931180; doi:10.1002/advs.202517442)
Supplement: Supplementary file 1 — Supporting Information [file ADVS-13-e17442-s001.docx]

Supporting Information

**Compartmentalized Homeostasis Drives High Bamboo Forest Productivity Under Nutrient Imbalance**

*Zhikang Wang, Quan Li, Man Shi, Marcio F.A. Leite, Xinli Chen, Eiko E. Kuramae, Viviane Cordovez, Tingting Cao, Chenglei Zhu, Libin Zhou, Wenjuan Yu, Zhiyao Tang, Changhui Peng, and Xinzhang Song^*^*


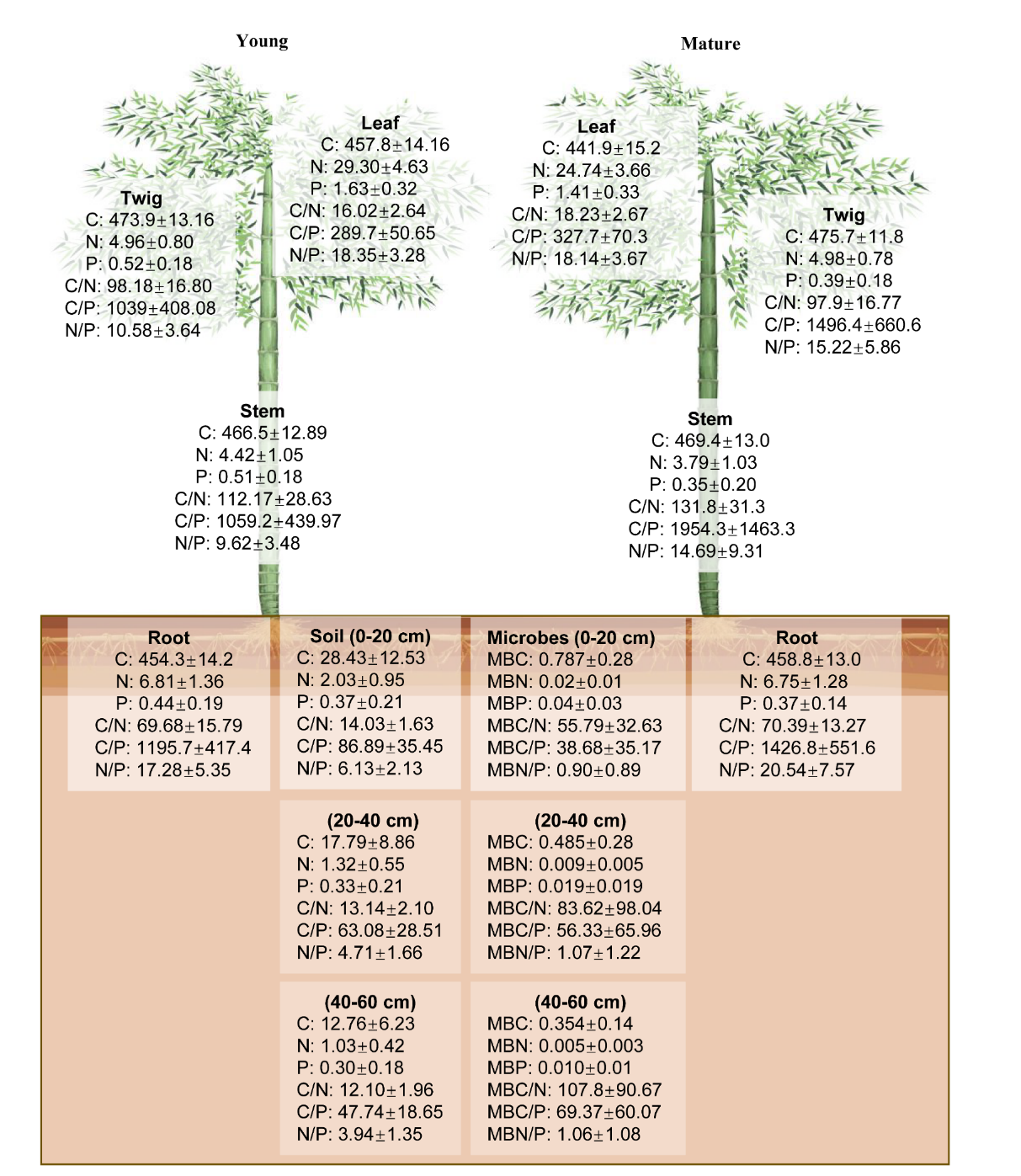


**Figure S1.** Overview of total carbon (C), nitrogen (N), and phosphorus (P) concentrations (g/kg) in plants, soils and microorganisms of Moso bamboo forests across subtropical regions. Plant data (n = 648) include leaves, twigs, stems, and roots for young (one year old) and mature bamboo (three years old). Soil data include soil (n = 243) and microbial biomass (n = 243) at three different depths (0-20cm, 20-40cm, and 40-60cm). All data are presented as mean ± SD from 27 sampling sites.


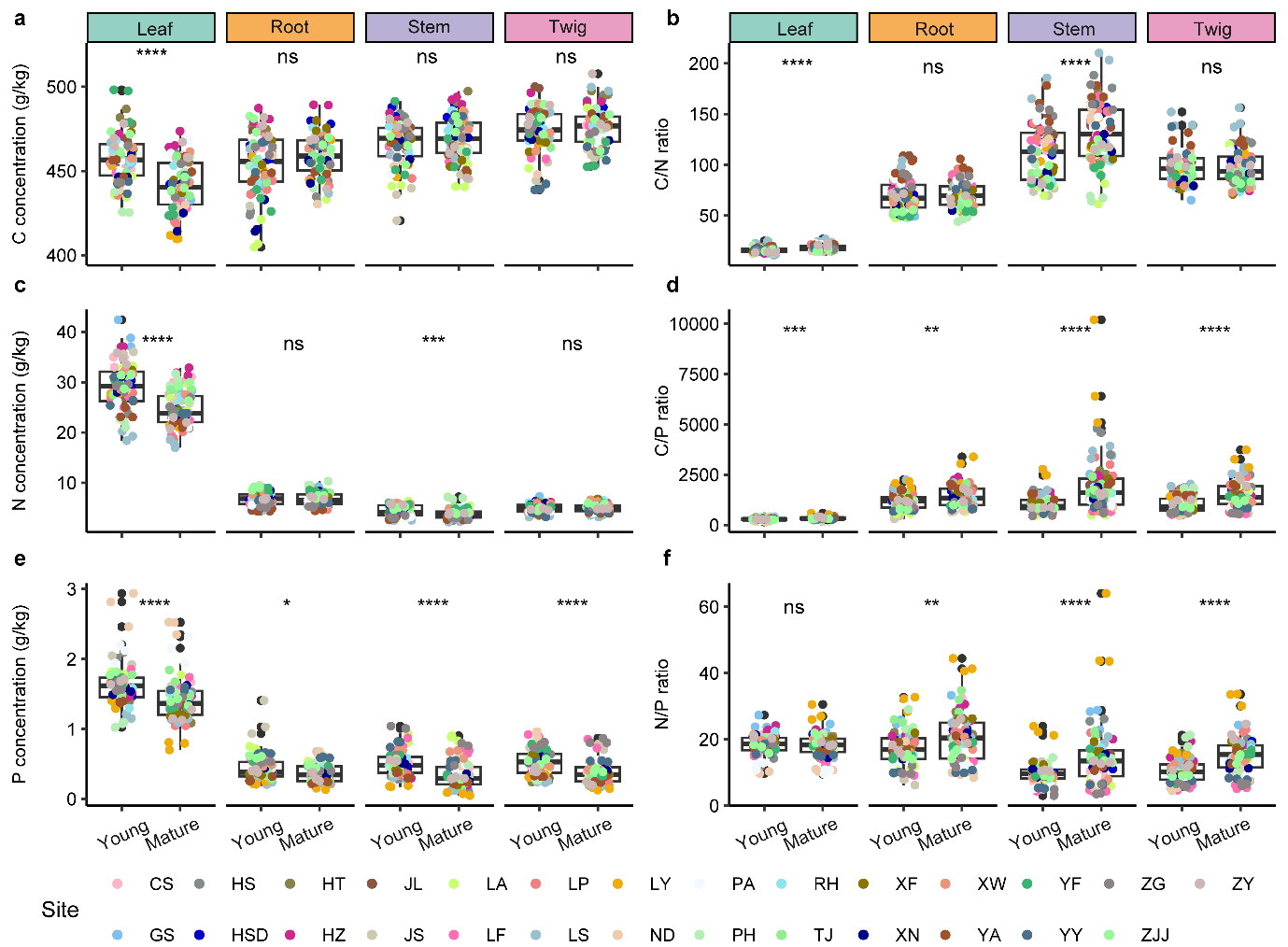


**Figure S2.** Plant C, N, and P concentrations and C/N ratio, C/P ratio, and N/P ratio in different tissue of Moso bamboo. Different tissues: leaf, root, stem, and twig. Age: one year old (young) and three years old (mature). One-way ANOVA was applied to test the significant difference of C, N, P, and their ratios between young and mature bamboos. Sites: CS (Chishui), GS (Guangshan), HS (Huangshan), HSD (Heishidu), HT (Huitong), HZ (Hezhou), JL (Jiaoling), JS (Jiangshan), LA (Linan), LF(Laifeng), LP (Liping), LS (Lishui), LY(Liyang), PA (Panan), PH (Pinghe), RH (Renhua), TJ (Taojiang), XF (Xinfeng), XN (Xianning), XW (Xingwen), YA (Yongan), YF (Yifeng), YY (Yuyao), ZG (Zigui), ZJJ (Zhangjiajie), ZY (Ziyuan).


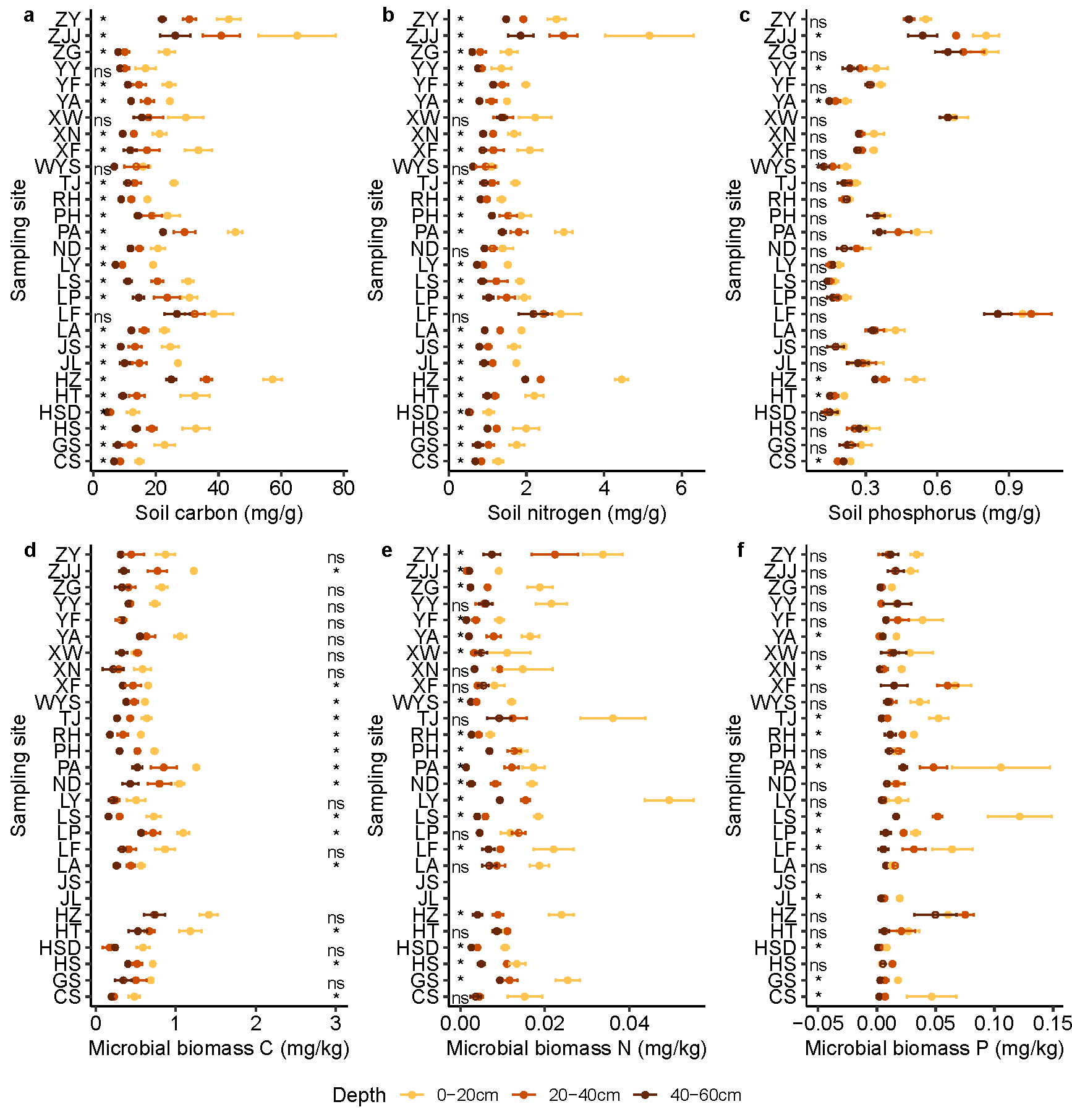


**Figure S3.** Soil C, N, and P and microbial biomass C, N, and P contents at different soil depth across all sampling sites in Moso bamboo forests. Sites: CS (Chishui), GS (Guangshan), HS (Huangshan), HSD (Heishidu), HT (Huitong), HZ (Hezhou), JL (Jiaoling), JS (Jiangshan), LA (Linan), LF(Laifeng), LP (Liping), LS (Lishui), LY(Liyang), PA (Panan), PH (Pinghe), RH (Renhua), TJ (Taojiang), XF (Xinfeng), XN (Xianning), XW (Xingwen), YA (Yongan), YF (Yifeng), YY (Yuyao), ZG (Zigui), ZJJ (Zhangjiajie), ZY (Ziyuan).


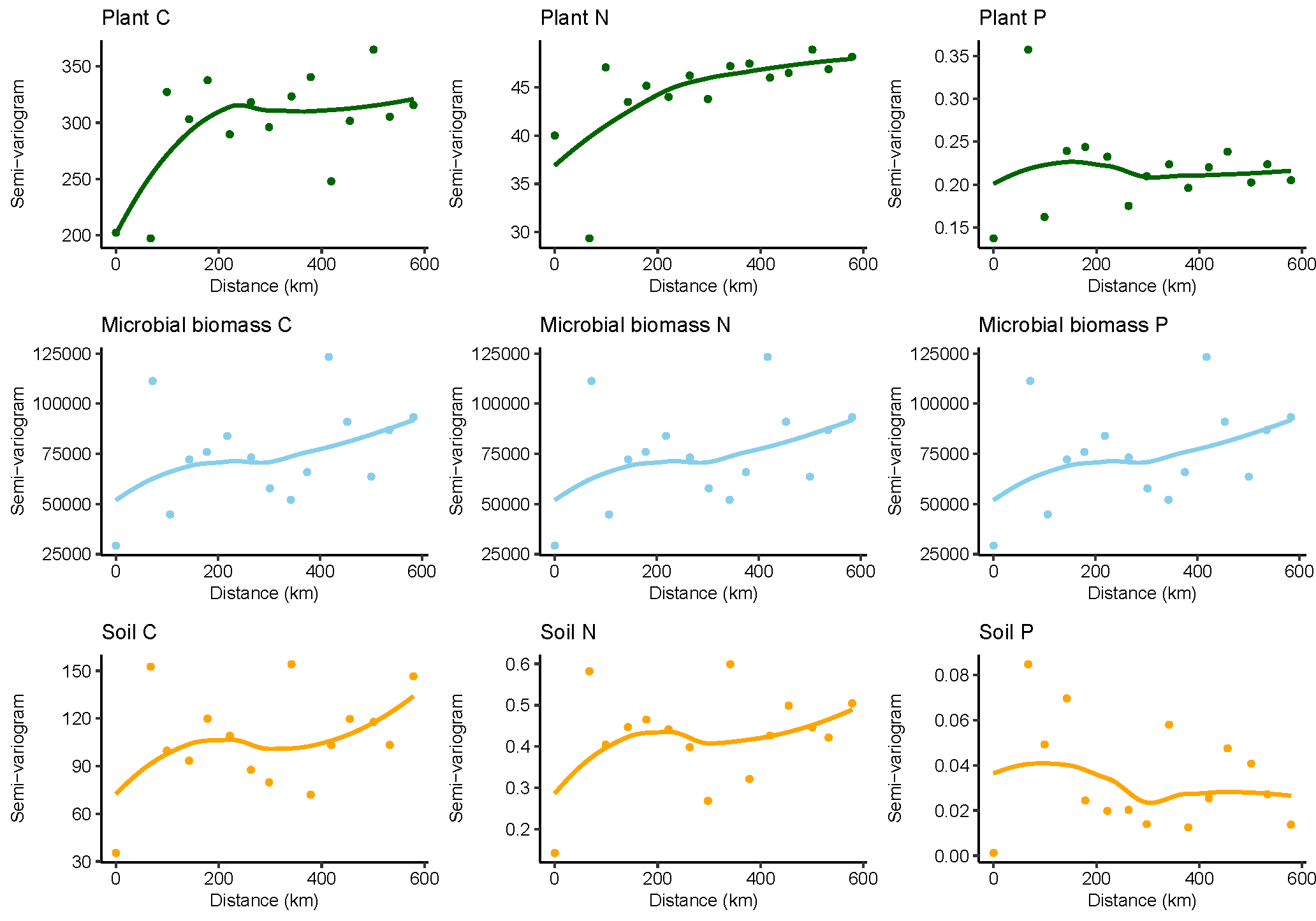


**Figure S4.** Empirical semi-variograms of elemental contents in plant-soil-microbe system. Microbial biomass variograms increase steeply and reach sill at very short distances, whereas plant element variograms increase more gradually and typically plateau at larger distances. Soil elements show variable behavior with some variables exhibiting large nugget components.


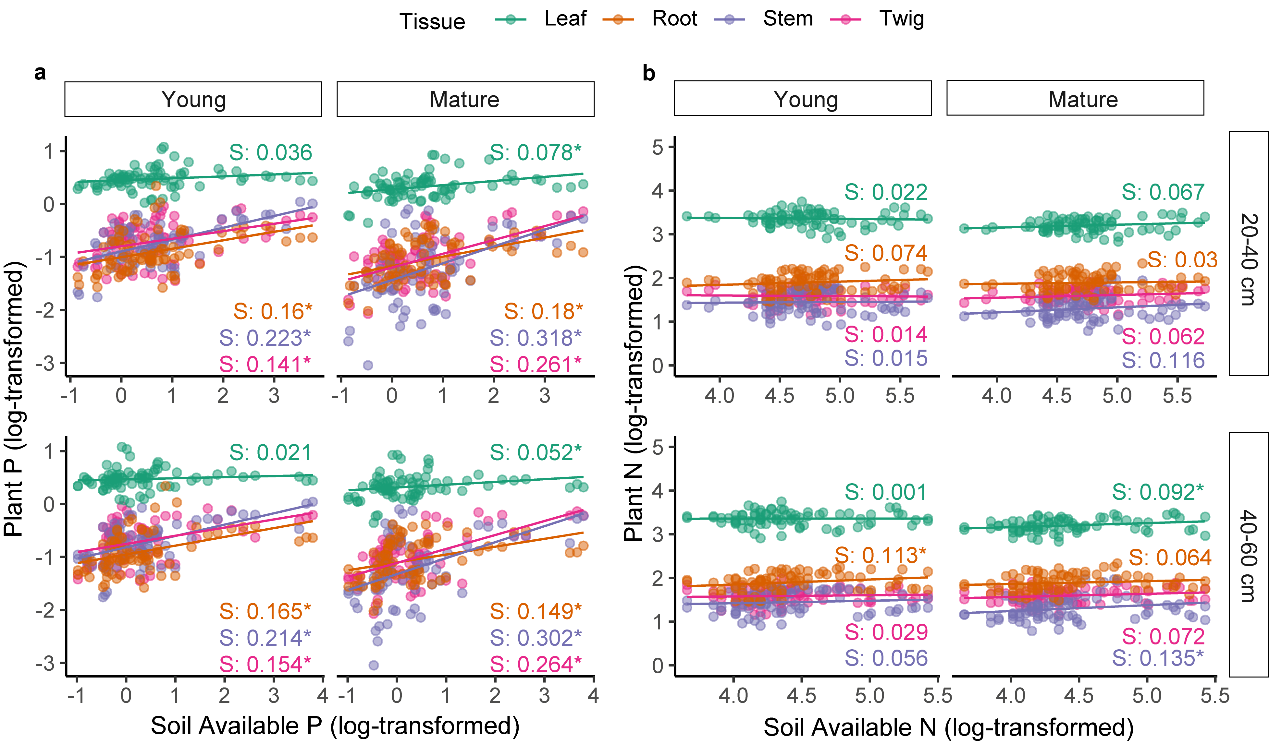


**Figure S5.** Plant P (a, *H_plantP_*) and N (b, *H_plantN_*) homeostasis in response to changes in soil available P and N, respectively. Plant P and N in different tissues (leaf, root, stem, and twig) for young (one year old) and mature (three years old) bamboo were compared upon soil available N and P at 20-40 cm and 40-60 cm. The slopes (S, namely, model coefficients) of the linear regression models indicate the *H_plantP(N)_* of different tissues (n **=** 81 for each regression). The data were transformed based on natural logarithms. The detailed information of the linear regression models is listed in supplemental **Table S2**.


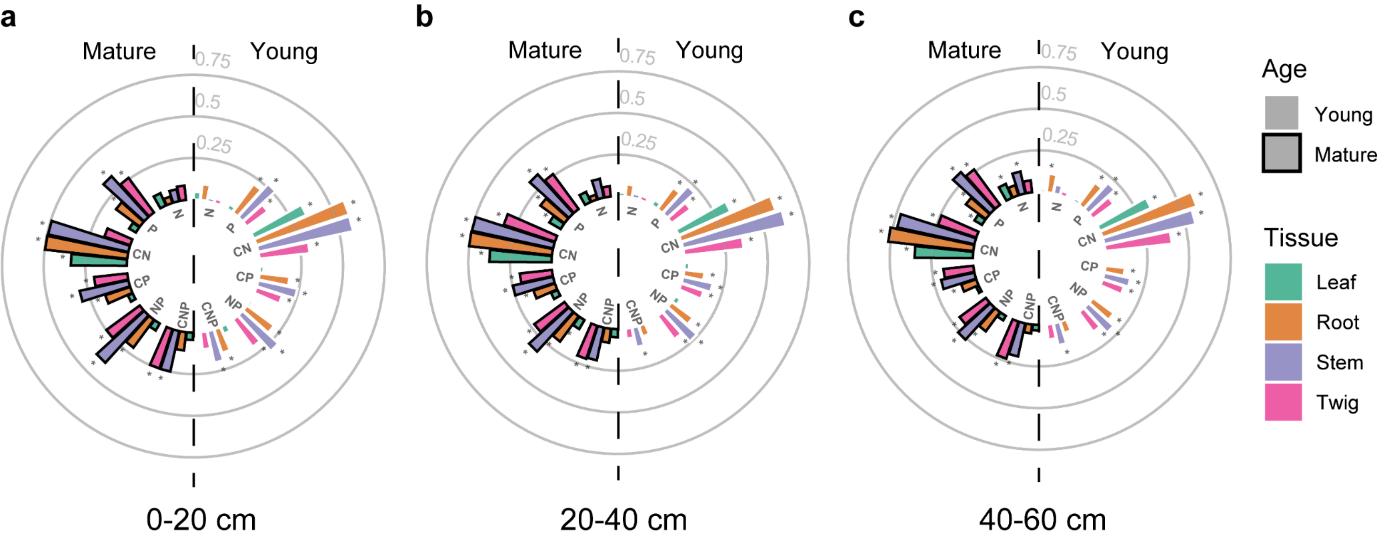


**Figure S6.** Homeostasis level of plant N, P, C:N, C:P, N:P, and C:N:P in leaves, roots, stems, and twigs for both young and mature bamboo. The values were extracted from the coefficients by fitting linear models between plant nutrients (or their ratios) and soil available nutrients (or their ratios) at three different depths (0-20cm, 20-40cm, and 40-60cm), respectively. The significances of the coefficients were test by the model and indicated as asterisk above the bars (*: *P* < 0.05). The bars on the left (with boarder line) indicates the homeostasis for mature bamboo, while the right one indicates homeostasis for younger bamboo.


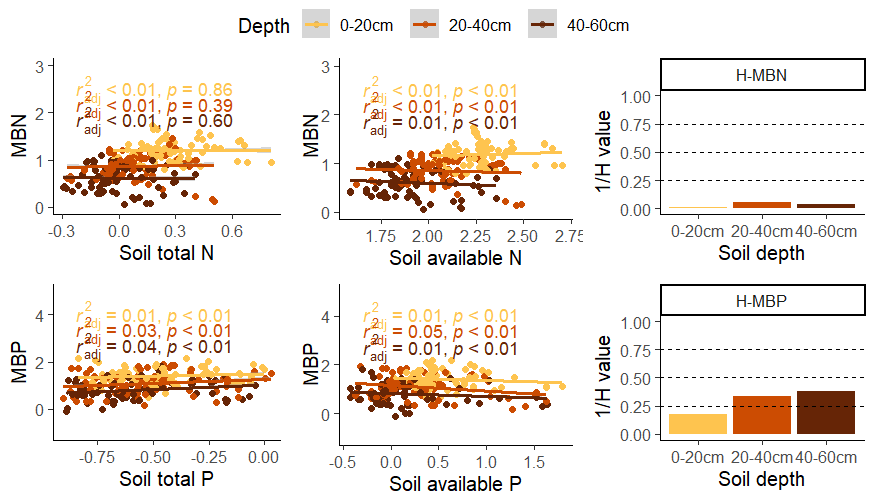


**Figure S7.** Stoichiometric homeostasis of microbial biomass N and P across different soil depths (0-20 cm, 20-40 cm, and 40-60 cm). The 1/*H* values were extracted from the coefficients by fitting linear models between microbial biomass N (MBN) or P (MBP) and soil total N and P at three different depths (0-20cm, 20-40cm, and 40-60cm), respectively. All datasets with significant regressions (*P* < 0.05) and 0<1/*H*<1were arbitrarily classified as: 0< 1/*H* <0.25 ‘homeostatic’, 0.25< 1/*H* <0.5 ‘weakly homeostatic’, 0.5< 1/*H* <0.75 ‘weakly plastic’, 1/*H* >0.75 ‘plastic’.


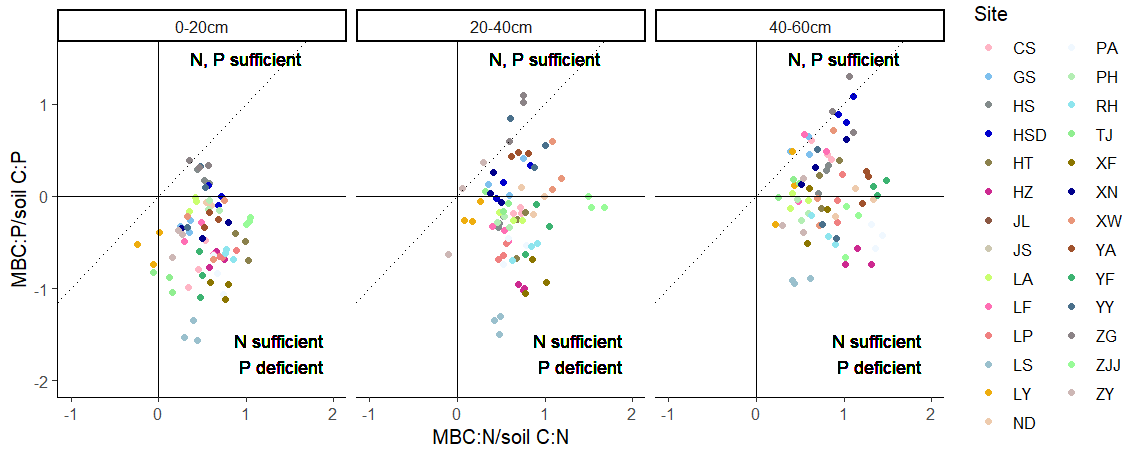


**Figure S8.** Microbial N, P limitation in relationship with soil C: N and C: P ratios at different depths. The points fall within the first quadrant indicate both N- and P-sufficient, and those points fall within the fourth quadrant indicate N sufficient and P deficient, according to the microbial homeostasis and flexibility model Asada et al ^[1]^. The information of all sites is listed in Table S5.

**Figure S9.** Internal N-P relationship of plants and microorganisms at all sampling sites in bamboo forests. **a.** Bamboo exhibits a stable relationship between internal N and P concentrations by linear mixed model (*R*^2^ > 0.8, *P* < 0.05). **b**. Linear regression relationship between soil microbial biomass N and P. The fitting lines are plotted by sites and the red dashed line represents the N-P relationship for all sites. The plant data are presented from all tissues and the microbial data are presented from all depth.


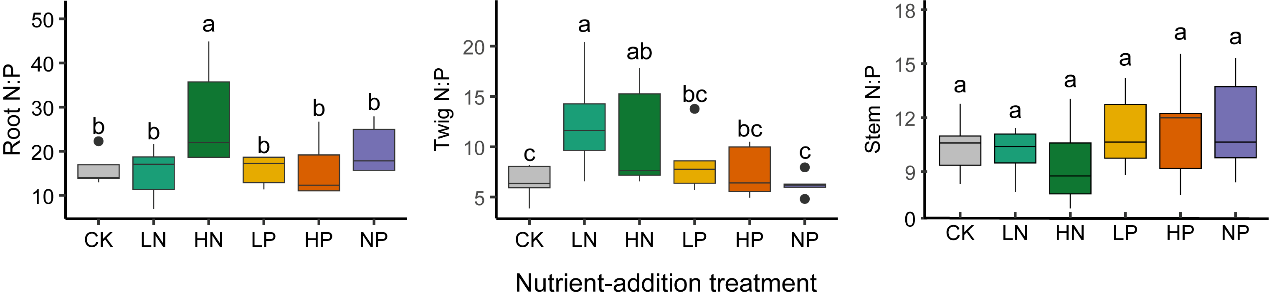


**Figure S10.** Plant-soil-microorganisms N:P ratio in response to different nutrient addition treatments. Significant differences between treatments were indicated by different letters above the boxes (*P* < 0.05). The treatments are: CK (no addition), HN (high N input), HP (high P input), LN (low N input), LP (low P input), and NP (N and P input).


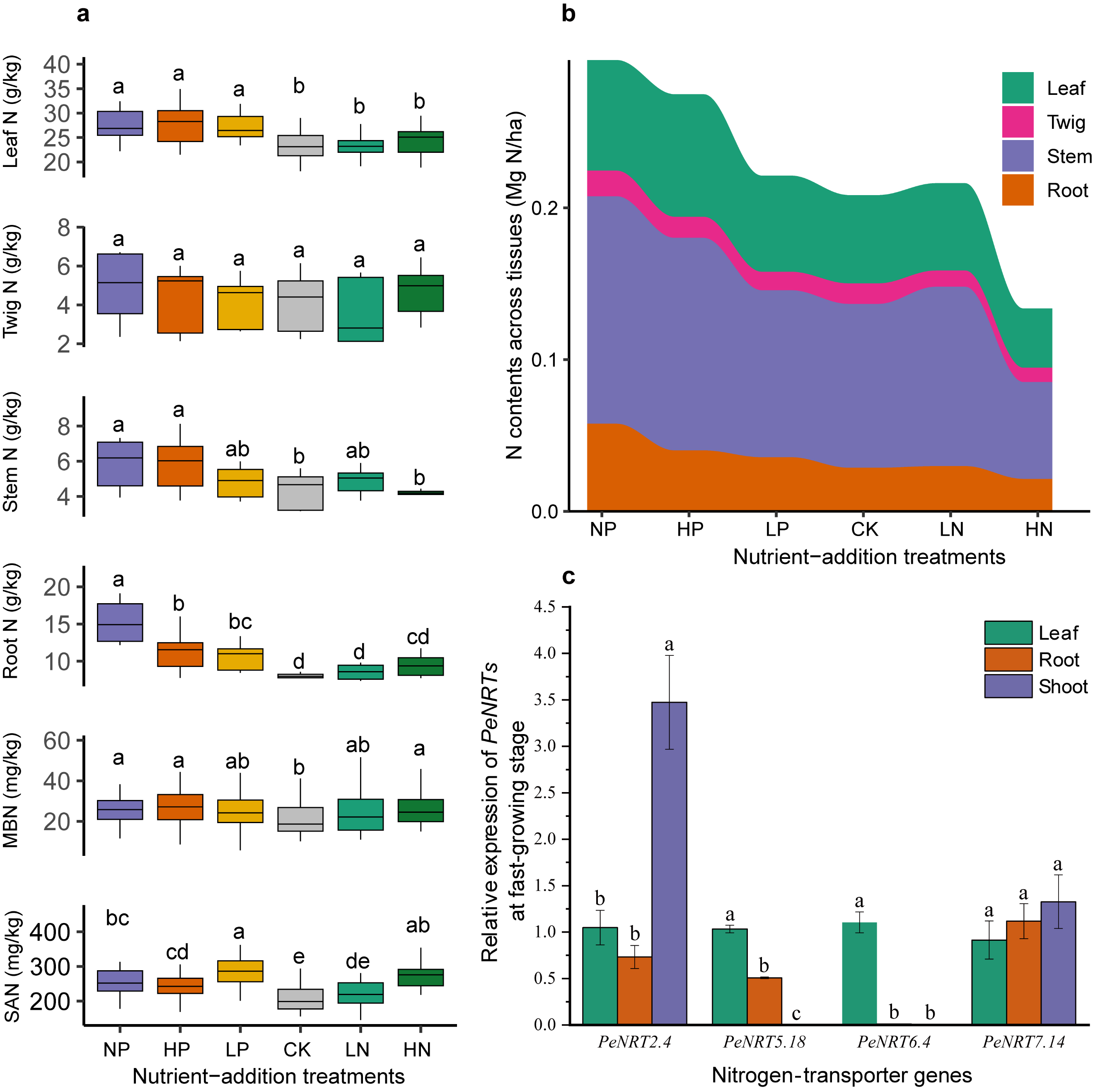


**Figure S11.** Effects of nutrient additions on N status in plant-soil-microbe system. (a) Total N concentrations in different plant tissues and microbial biomass N under different nutrient-addition treatments. (b) Allocation N contents (Mg N ha^-1^) across different tissues in nutrient-addition experiment. (c) *PeNRT* (nitrogen-transporter genes) relative expression across leaf, root, and shoot tissues during the fast-growing stage of bamboo. The treatments are: CK (no addition), HN (high N input), HP (high P input), LN (low N input), LP (low P input), and NP (N and P input).


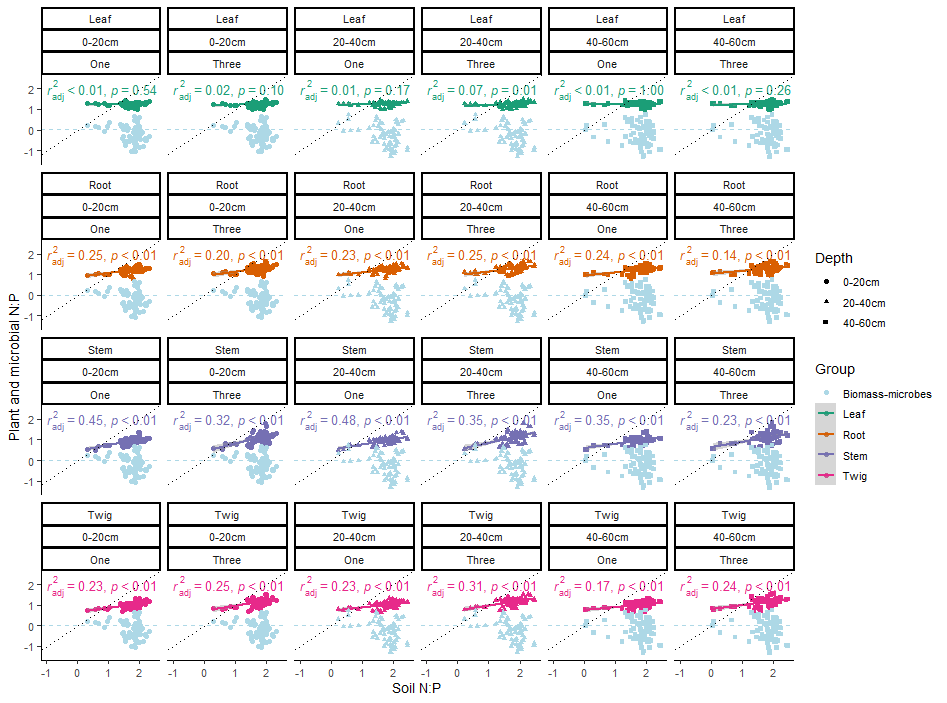


**Figure S12.** Correlations between plant’s N:P, microbial N:P and soil N:P by incorporating the factors of plant tissue, age, and soil depth. Note that both axes have a logarithmic scale.


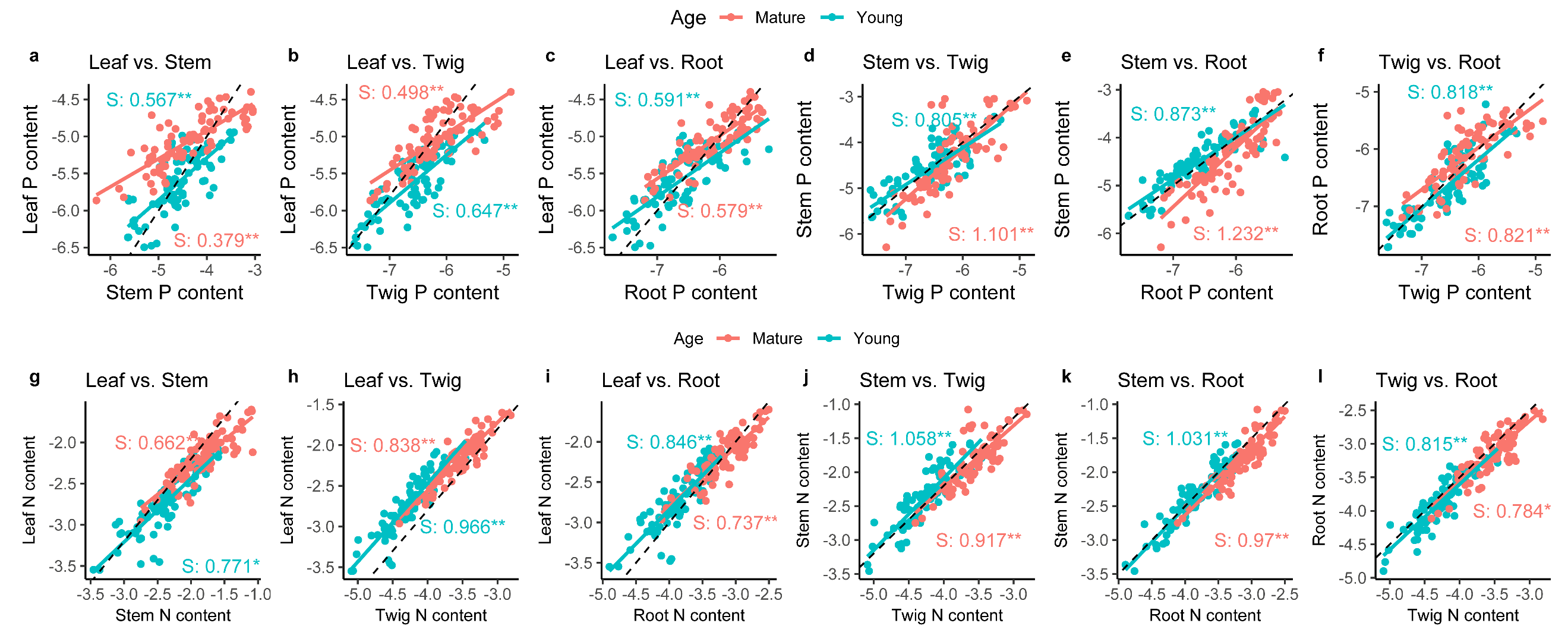


**Figure S13.** Allocation pattern of P (a-f) and N (g-l) contents across different tissues in young and mature bamboo. The correlations were analyzed by comparing P(N) contents in leaf vs. stem, leaf vs. twig, leaf vs. root, stem vs. twig, stem vs. root, and root vs. twig. P(N) contents were calculated for all sampling sites. A scaling approach, log(Y) = a + b × log(X), was applied to explore the allocation of P(N) in different tissues. A slope (b) smaller than 1 indicates slower changes of P(N) in Y than in X. A likelihood ratio test was used to assess the heterogeneity between the reduced major axis (RMA) regression slopes of different groups ^[2]^. Note that both axes have a logarithmic scale.


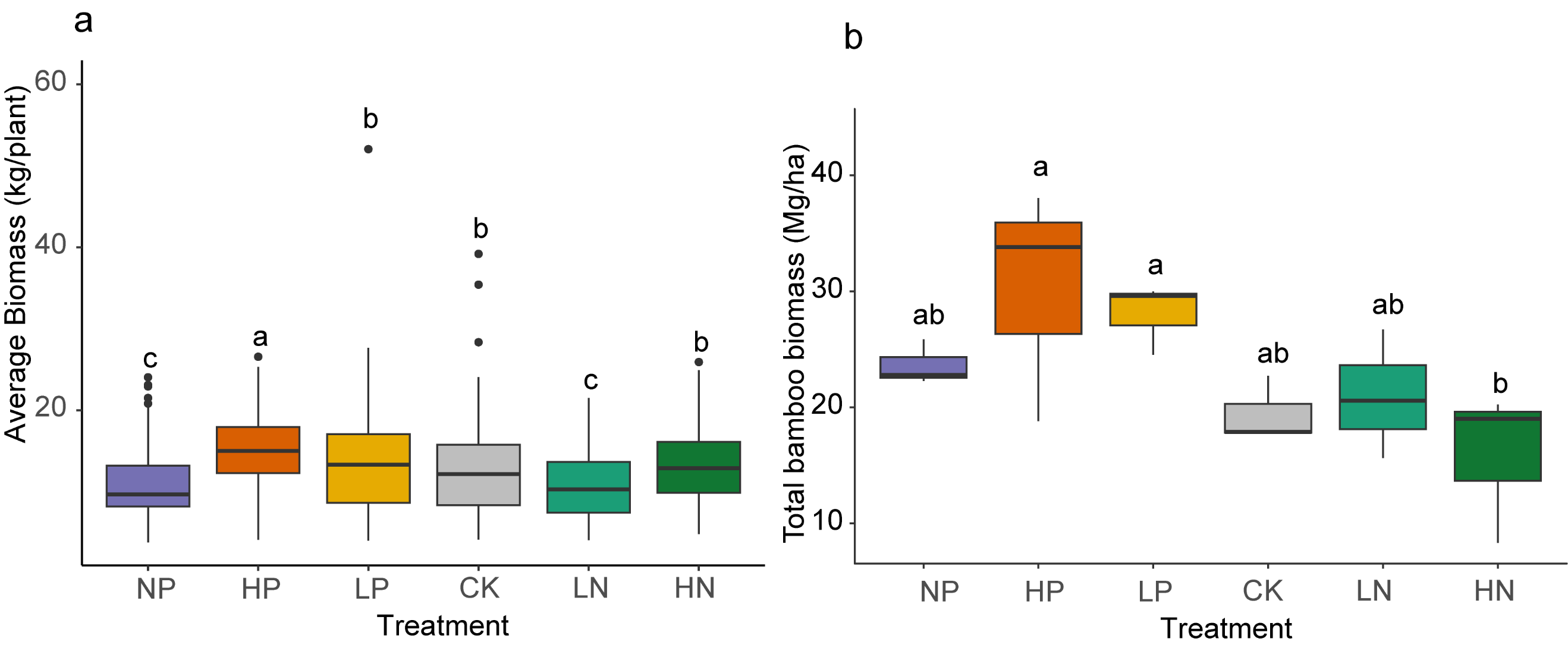


**Figure S14.** Effects of nutrient additions on bamboo biomass at individual (a, kg plant^-1^) ecosystem level (b, Mg ha^-1^). The differences of biomass between treatments were analyzed using one-way ANOVA. The numbers of individual culms are 184, 146, 241, 235, 248, and 258 for treatments CK (no addition), HN (high N input), HP (high P input), LN (low N input), LP (low P input), and NP (N and P input), respectively.


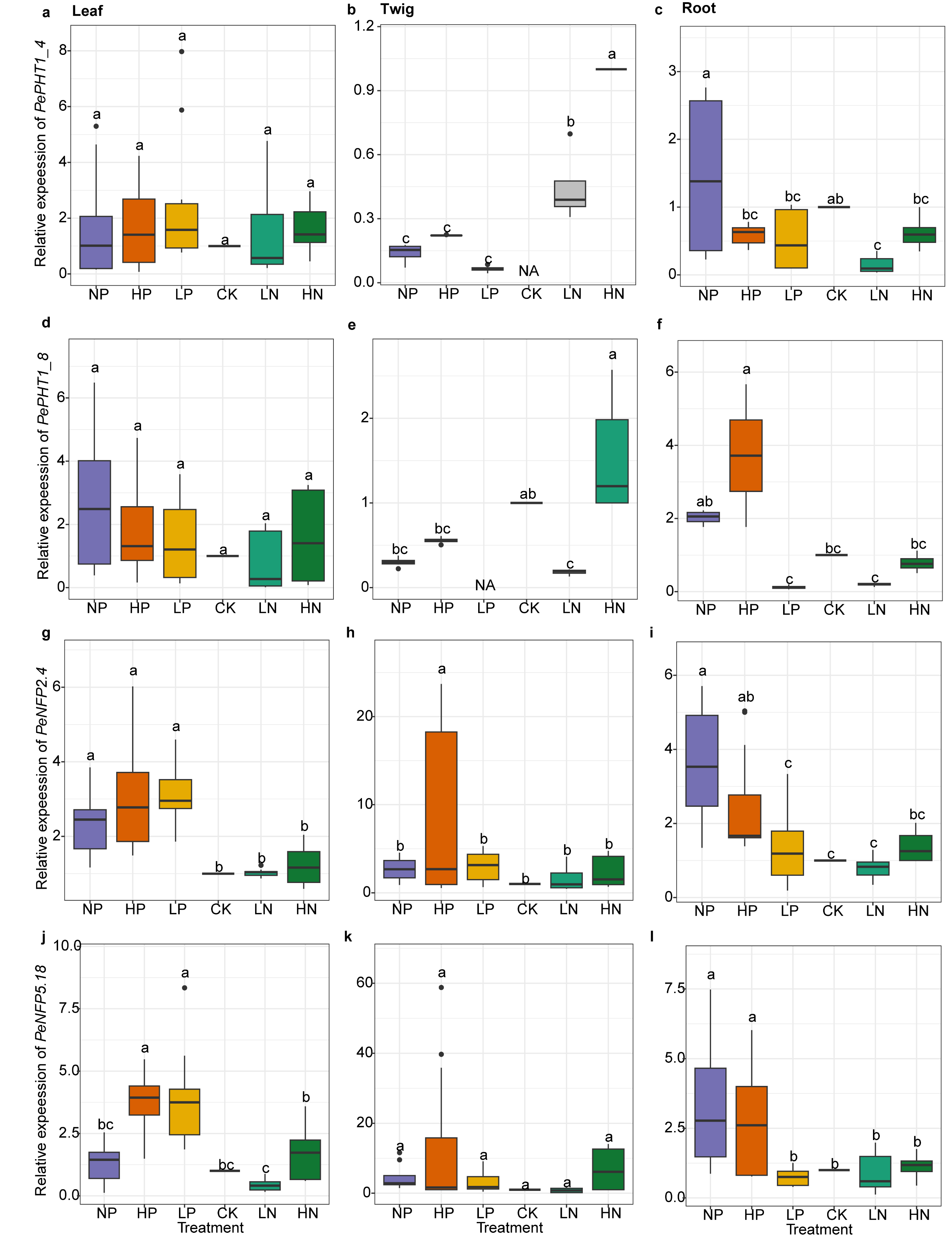


**Figure S15.** Responses of relative expression of P-transporters (*PePHT1-4* and *PePHT1-8*) and N-transporters (*PeNRT2.4* and *PeNRT5.18*) in different tissues (leaf, twig, and root) to nutrient additions. Significant differences between treatments were indicated by different letters above the boxes (*P* < 0.05). NA indicates ‘not detected’. The RNA concentration in the stem was hardly detected due to lignification. The treatments are: CK (no addition), HN (high N input), HP (high P input), LN (low N input), LP (low P input), and NP (N and P input).

**
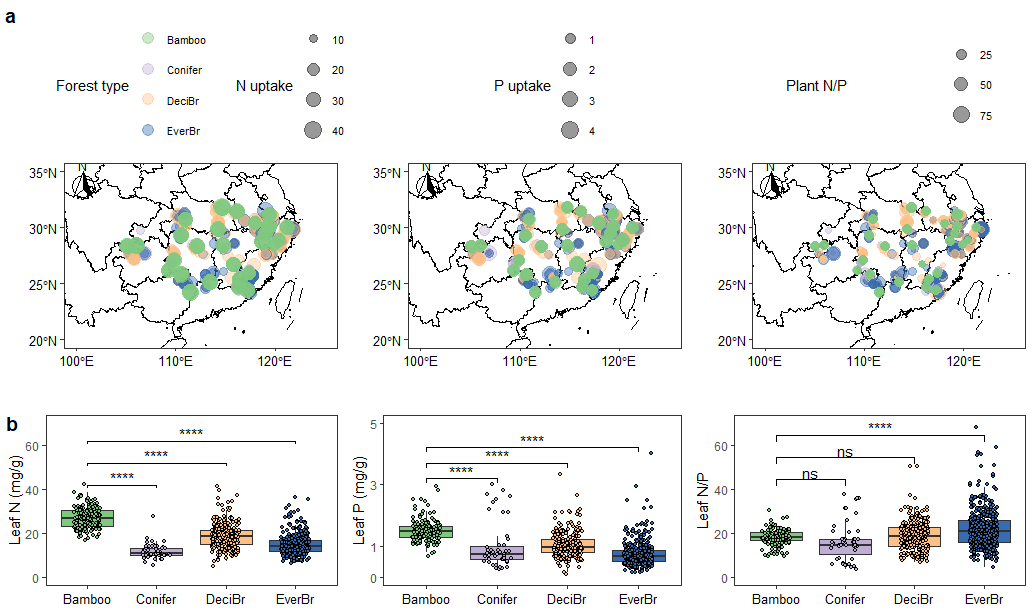
**

**Figure S16.** Comparison of leaf N, P concentrations (mg/g) and N:P ratios between bamboo and other tree species sourced from 27 independent sites. To minimize the effect of climate and geography on the results, we selected other forest types that near to Bamboo sampling locations (± 1° longitude or altitude). The significances were presented in the main figure. The source data of plant N and P was obtained from Tian et al., 2024.^[3]^

**
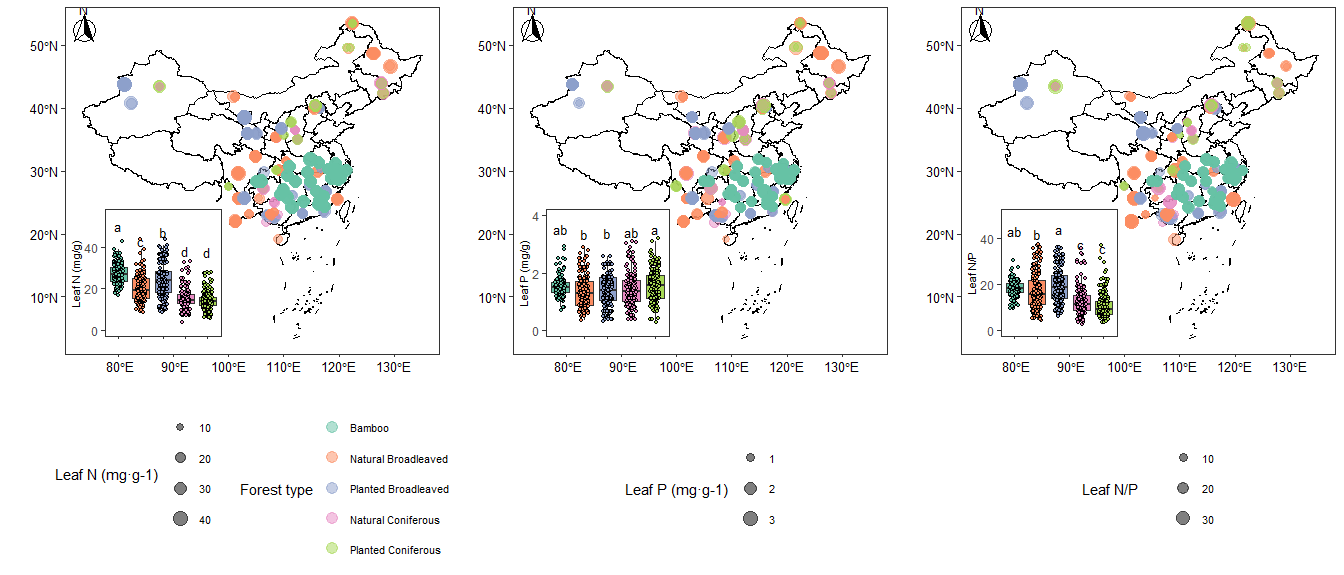
**

**Figure S17.** Leaf N, P and N/P ratio in bamboo forests, natural broadleaved forest, planted broadleaved forest, natural coniferous forest, and planted coniferous forest. Bamboo exhibits relatively higher N and competitive P concentrations compared to other forests. The data was obtained from field investigation in this study and previous published study by Gong et al.^[4]^


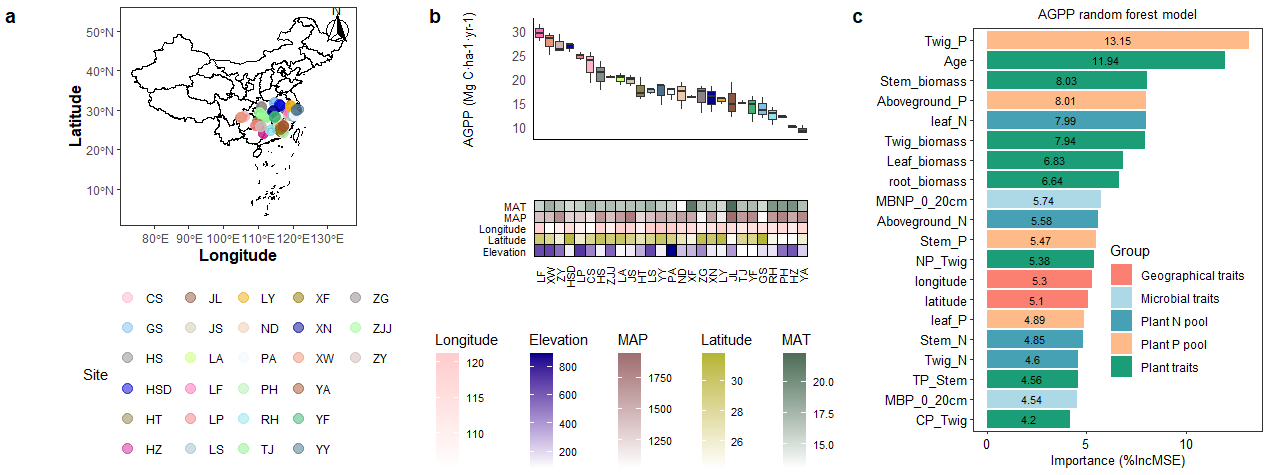


**Figure S18.** Variation of AGPP across sites and the underlying predictors. a. Sampling sites across subtropical forests. b. The potential correlations between AGPP in bamboo forests and geographical traits. c. Random Forest analysis for revealing the underlying factors for driving AGPP variation. Sites: CS (Chishui), GS (Guangshan), HS (Huangshan), HSD (Heishidu), HT (Huitong), HZ (Hezhou), JL (Jiaoling), JS (Jiangshan), LA (Linan), LF(Laifeng), LP (Liping), LS (Lishui), LY (Liyang), PA (Panan), PH (Pinghe), RH (Renhua), TJ (Taojiang), XF (Xinfeng), XN (Xianning), XW (Xingwen), YA (Yongan), YF (Yifeng), YY (Yuyao), ZG (Zigui), ZJJ (Zhangjiajie), ZY (Ziyuan). MAT: mean annual temperature. MAP: mean annual precipitation.

**Table S1** The detailed information of the linear regression models for Homeostasis analyses for plant P and N corresponding to the changes in soil available P and N, respectively.

| **Relationship** | **Depth** | **Age** | **Tissue** | **Intercept** | **Coefficients** | ***R^2^*** | ***P*-value** | **Homeostasis** |
| --- | --- | --- | --- | --- | --- | --- | --- | --- |
| **Plant P vs Soil available P** | 0-20cm | Young | Leaf | 0.434 | 0.031 | 0.018 | 0.239 | 0.031 |
|  |  |  | Root | -1.158 | 0.213 | 0.210 | 0.000 | 0.213 |
|  |  |  | Stem | -1.074 | 0.268 | 0.328 | 0.000 | 0.268 |
|  |  |  | Twig | -0.893 | 0.155 | 0.115 | 0.002 | 0.155 |
|  |  | Mature | Leaf | 0.271 | 0.047 | 0.028 | 0.143 | 0.047 |
|  |  |  | Root | -1.271 | 0.177 | 0.141 | 0.001 | 0.177 |
|  |  |  | Stem | -1.630 | 0.331 | 0.191 | 0.000 | 0.331 |
|  |  |  | Twig | -1.343 | 0.258 | 0.230 | 0.000 | 0.258 |
|  | 20-40cm | Young | Leaf | 0.448 | 0.036 | 0.036 | 0.094 | 0.036 |
|  |  |  | Root | -1.004 | 0.160 | 0.171 | 0.000 | 0.16 |
|  |  |  | Stem | -0.895 | 0.223 | 0.331 | 0.000 | 0.223 |
|  |  |  | Twig | -0.797 | 0.141 | 0.138 | 0.001 | 0.141 |
|  |  | Mature | Leaf | 0.277 | 0.078 | 0.114 | 0.002 | 0.078 |
|  |  |  | Root | -1.174 | 0.180 | 0.211 | 0.000 | 0.18 |
|  |  |  | Stem | -1.436 | 0.318 | 0.255 | 0.000 | 0.318 |
|  |  |  | Twig | -1.200 | 0.261 | 0.341 | 0.000 | 0.261 |
|  | 40-60cm | Young | Leaf | 0.465 | 0.021 | 0.012 | 0.341 | 0.021 |
|  |  |  | Root | -0.952 | 0.165 | 0.188 | 0.000 | 0.165 |
|  |  |  | Stem | -0.817 | 0.214 | 0.311 | 0.000 | 0.214 |
|  |  |  | Twig | -0.753 | 0.154 | 0.167 | 0.000 | 0.154 |
|  |  | Mature | Leaf | 0.311 | 0.052 | 0.052 | 0.045 | 0.052 |
|  |  |  | Root | -1.103 | 0.149 | 0.148 | 0.000 | 0.149 |
|  |  |  | Stem | -1.324 | 0.302 | 0.236 | 0.000 | 0.302 |
|  |  |  | Twig | -1.112 | 0.264 | 0.357 | 0.000 | 0.264 |
| **Plant N vs Soil available N** | 0-20cm | Young | Leaf | 3.115 | 0.046 | 0.009 | 0.413 | 0.046 |
|  |  |  | Root | 1.395 | 0.096 | 0.022 | 0.192 | 0.096 |
|  |  |  | Stem | 1.482 | -0.007 | 0.000 | 0.934 | 0.007 |
|  |  |  | Twig | 1.440 | 0.028 | 0.003 | 0.635 | 0.028 |
|  |  | Mature | Leaf | 2.779 | 0.081 | 0.031 | 0.120 | 0.081 |
|  |  |  | Root | 1.677 | 0.041 | 0.005 | 0.548 | 0.041 |
|  |  |  | Stem | 0.916 | 0.073 | 0.009 | 0.409 | 0.073 |
|  |  |  | Twig | 1.141 | 0.087 | 0.031 | 0.122 | 0.087 |
|  | 20-40cm | Young | Leaf | 3.462 | -0.022 | 0.003 | 0.646 | 0.022 |
|  |  |  | Root | 1.545 | 0.074 | 0.019 | 0.231 | 0.074 |
|  |  |  | Stem | 1.373 | 0.015 | 0.001 | 0.832 | 0.015 |
|  |  |  | Twig | 1.657 | -0.014 | 0.001 | 0.776 | 0.014 |
|  |  | Mature | Leaf | 2.885 | 0.067 | 0.030 | 0.128 | 0.067 |
|  |  |  | Root | 1.747 | 0.030 | 0.004 | 0.597 | 0.03 |
|  |  |  | Stem | 0.748 | 0.116 | 0.032 | 0.118 | 0.116 |
|  |  |  | Twig | 1.300 | 0.062 | 0.023 | 0.190 | 0.062 |
|  | 40-60cm | Young | Leaf | 3.353 | 0.001 | 0.000 | 0.980 | 0.001 |
|  |  |  | Root | 1.399 | 0.113 | 0.050 | 0.048 | 0.113 |
|  |  |  | Stem | 1.198 | 0.056 | 0.009 | 0.403 | 0.056 |
|  |  |  | Twig | 1.462 | 0.029 | 0.005 | 0.540 | 0.029 |
|  |  | Mature | Leaf | 2.798 | 0.092 | 0.066 | 0.023 | 0.092 |
|  |  |  | Root | 1.609 | 0.064 | 0.019 | 0.227 | 0.064 |
|  |  |  | Stem | 0.703 | 0.135 | 0.050 | 0.049 | 0.135 |
|  |  |  | Twig | 1.278 | 0.072 | 0.035 | 0.101 | 0.072 |

**Table S2** Slopes of reduced major axis (RMA) regression among tissues for the content of N and P across different tissues

| **Age** | **Comparison** | **Slope** | **Lower-CI** | **Upper-CI** | **P value** | **R-squared** |
| --- | --- | --- | --- | --- | --- | --- |
| Mature | leaf_P vs Stem_P | 0.379 | 0.315 | 0.442 | 3.7935004E-19 | 0.64 |
| Young | leaf_P vs Stem_P | 0.567 | 0.456 | 0.679 | 5.9342782E-16 | 0.57 |
| Mature | leaf_P vs Twig_P | 0.498 | 0.405 | 0.591 | 6.0907732E-17 | 0.59 |
| Young | leaf_P vs Twig_P | 0.647 | 0.555 | 0.739 | 4.6316296E-23 | 0.71 |
| Mature | leaf_P vs Root_P | 0.579 | 0.497 | 0.661 | 3.0620037E-23 | 0.72 |
| Young | leaf_P vs Root_P | 0.591 | 0.487 | 0.696 | 4.3452834E-18 | 0.62 |
| Mature | Stem_P vs Twig_P | 1.101 | 0.918 | 1.284 | 1.7857756E-19 | 0.65 |
| Young | Stem_P vs Twig_P | 0.805 | 0.666 | 0.944 | 1.4113523E-18 | 0.63 |
| Mature | Stem_P vs Root_P | 1.232 | 1.063 | 1.401 | 5.5915901E-24 | 0.73 |
| Young | Stem_P vs Root_P | 0.873 | 0.765 | 0.981 | 1.4691456E-26 | 0.76 |
| Mature | Root_P vs Twig_P | 0.737 | 0.604 | 0.871 | 1.3121291E-17 | 0.61 |
| Young | Root_P vs Twig_P | 0.848 | 0.722 | 0.974 | 5.3900119E-22 | 0.69 |
| Mature | leaf_N vs Stem_N | 0.662 | 0.562 | 0.762 | 1.2054376E-21 | 0.69 |
| Young | leaf_N vs Stem_N | 0.771 | 0.674 | 0.868 | 2.9559525E-26 | 0.76 |
| Mature | leaf_N vs Twig_N | 0.838 | 0.754 | 0.922 | 1.8466306E-32 | 0.83 |
| Young | leaf_N vs Twig_N | 0.966 | 0.862 | 1.069 | 1.2980248E-30 | 0.81 |
| Mature | leaf_N vs Root_N | 0.737 | 0.640 | 0.833 | 4.6807749E-25 | 0.74 |
| Young | leaf_N vs Root_N | 0.846 | 0.736 | 0.956 | 2.2499226E-25 | 0.75 |
| Mature | Stem_N vs Twig_N | 0.917 | 0.761 | 1.073 | 5.5012552E-19 | 0.64 |
| Young | Stem_N vs Twig_N | 1.058 | 0.926 | 1.190 | 1.8649454E-26 | 0.76 |
| Mature | Stem_N vs Root_N | 0.970 | 0.868 | 1.071 | 2.9755557E-31 | 0.82 |
| Young | Stem_N vs Root_N | 1.031 | 0.940 | 1.121 | 1.9113203E-36 | 0.87 |
| Mature | Root_N vs Twig_N | 0.906 | 0.777 | 1.036 | 5.6973827E-23 | 0.71 |
| Young | Root_N vs Twig_N | 0.977 | 0.866 | 1.087 | 4.9330599E-29 | 0.80 |

Note: CI indicates confidence interval

**Table S3** Comparison of leaf N:P standard deviations of bamboo and other plant species in forests across 27 sampling sites

| No. | Site | Species | Leaf N:P (SD) | No. | Site | Species | Leaf N:P (SD) |
| --- | --- | --- | --- | --- | --- | --- | --- |
| 1 | CS | *Phyllostachys edulis* | 1.40 | 83 | YA | *Camellia sinensis* | 2.29 |
| 2 | CS | *Cupressus funebris* | 1.55 |  |  |  |  |
| 3 | GS | *Phyllostachys edulis* | 2.04 | 84 | YA | *Citrus grandis* | 0.59 |
| 4 | GS | *Celtis julianae* | 13.40 | 85 | YA | *Cleyera japonica* | 1.73 |
| 5 | GS | *Quercus fabri* | 1.79 | 86 | YA | *Cunninghamia lanceolata* | 5.86 |
| 6 | HS | *Phyllostachys edulis* | 2.12 | 87 | YA | *Dendrocalamopsis oldhami* | 2.10 |
| 7 | HS | *Dalbergia hupeana* | 1.80 | 88 | YA | *Loropetalum chinense* | 1.06 |
| 8 | HS | *Eurya brevistyla* | 2.76 | 89 | YA | *Rhaphiolepis indica* | 6.07 |
| 9 | HS | *Euscaphis japonica (Thunb.) Dippel* | 0.44 | 90 | YA | *Vaccinium bracteatum* | 0.16 |
| 10 | HS | *Loropetalum chinense* | 3.30 | 91 | YA | *Viburnum erubescens* | 0.08 |
| 11 | HS | *Pinus massoniana* | 0.35 | 92 | YF | *Phyllostachys edulis* | 0.68 |
| 12 | HS | *Quercus fabri* | 2.37 | 93 | YF | *Castanopsis sclerophylla* | 8.73 |
| 13 | HS | *Quercus serrata* | 2.74 | 94 | YF | *Loropetalum chinense* | 1.23 |
| 14 | HS | *Rhododendron mariesii* | 0.94 | 95 | YF | *Pinus massoniana* | 1.02 |
| 15 | HS | *Rhododendron simsii* | 0.93 | 96 | YF | *Vaccinium bracteatum* | 8.14 |
| 16 | HS | *Rhus chinensis* | 1.74 | 97 | YY | *Alniphyllum fortunei* | 2.09 |
| 17 | HSD | *Phyllostachys edulis* | 0.59 | 98 | YY | *Phyllostachys edulis* | 1.40 |
| 18 | HSD | *Quercus aliena* | 3.03 | 99 | YY | *Camellia fraterna* | 6.02 |
| 19 | HT | *Phyllostachys edulis* | 1.28 | 100 | YY | *Camellia japonica* | 4.14 |
| 20 | HT | *Cunninghamia lanceolata* | 3.91 | 101 | YY | *Carpinus londoniana* | 3.05 |
| 21 | HZ | *Phyllostachys edulis* | 0.76 | 102 | YY | *Castanopsis carlesii* | 4.43 |
| 22 | HZ | *Castanopsis fargesii* | 2.51 | 103 | YY | *Castanopsis fargesii* | 6.06 |
| 23 | HZ | *Pyracantha fortuneana* | 15.68 | 104 | YY | *Castanopsis sclerophylla* | 7.06 |
| 24 | JL | *Phyllostachys edulis* | 0.57 | 105 | YY | *Clerodendrum cyrtophyllum* | 3.62 |
| 25 | JL | *Cleyera japonica* | 2.88 | 106 | YY | *Cleyera japonica* | 2.86 |
| 26 | JL | *Rhaphiolepis indica* | 2.40 | 107 | YY | *Cunninghamia lanceolata* | 0.87 |
| 27 | JL | *Rhodomyrtus tomentosa* | 4.55 | 108 | YY | *Cyclobalanopsis gracilis* | 6.14 |
| 28 | JS | *Phyllostachys edulis* | 0.77 | 109 | YY | *Cyclobalanopsis myrsinifolia* | 3.50 |
| 29 | JS | *Camellia oleifera* | 2.75 | 110 | YY | *Cyclobalanopsis stewardiana* | 1.58 |
| 30 | LA | *Phyllostachys edulis* | 0.94 | 111 | YY | *Daphniphyllum macropodum* | 3.39 |
| 31 | LA | *Bennettiodendron leprosipes* | 10.90 | 112 | YY | *Distylium myricoides* | 1.94 |
| 32 | LA | *Cleyera japonica* | 0.91 | 113 | YY | *Elaeocarpus decipiens* | 6.42 |
| 33 | LA | *Gardenia jasminoides* | 3.10 | 114 | YY | *Eurya groffii* | 4.42 |
| 34 | LA | *Loropetalum chinense* | 3.72 | 115 | YY | *Eurya rubiginosa var. attenuata* | 10.39 |
| 35 | LA | *Quercus serrata* | 2.46 | 116 | YY | *Gardenia jasminoides* | 3.36 |
| 36 | LA | *Rhaphiolepis indica* | 1.27 | 117 | YY | *Glochidion puberum* | 3.18 |
| 37 | LA | *Rhododendron simsii* | 0.42 | 118 | YY | *Helicia cochinchinensis* | 7.83 |
| 38 | LA | *Trema cannabina* | 3.18 | 119 | YY | *Ilex rotunda* | 2.61 |
| 39 | LA | *Vaccinium bracteatum* | 4.19 | 120 | YY | *Illicium lanceolatum* | 2.64 |
| 40 | LF | *Phyllostachys edulis* | 1.22 | 121 | YY | *Liquidambar formosana* | 3.96 |
| 41 | LF | *Loropetalum chinense* | 5.08 | 122 | YY | *Lithocarpus glaber* | 4.66 |
| 42 | LF | *Quercus glauca* | 2.45 | 123 | YY | *Lithocarpus henryi* | 4.24 |
| 43 | LF | *Rhododendron simsii* | 0.64 | 124 | YY | *Litsea elongata* | 1.58 |
| 44 | LP | *Phyllostachys edulis* | 0.48 | 125 | YY | *Machilus thunbergii* | 3.01 |
| 45 | LP | *Cunninghamia lanceolata* | 3.91 | 126 | YY | *Myrica rubra* | 11.44 |
| 46 | LS | *Phyllostachys edulis* | 0.95 | 127 | YY | *Neolitsea aurata* | 4.20 |
| 47 | LS | *Camellia oleifera* | 2.75 | 128 | YY | *Neolitsea aurata var. chekiangensis* | 6.61 |
| 48 | LS | *Loropetalum chinense* | 1.24 | 129 | YY | *Ormosia henryi* | 2.50 |
| 49 | LS | *Rhododendron simsii* | 1.15 | 130 | YY | *Photinia serrulata* | 1.00 |
| 50 | LY | *Phyllostachys edulis* | 3.97 | 131 | YY | *Phyllanthus glaucus* | 0.23 |
| 51 | LY | *Clerodendrum cyrtophyllum* | 0.73 | 132 | YY | *Quercus acutissima* | 0.99 |
| 52 | LY | *Ilex chinensis* | 1.35 | 133 | YY | *Quercus fabri* | 4.45 |
| 53 | LY | *Loropetalum chinense* | 1.42 | 134 | YY | *Rhododendron ovatum* | 7.55 |
| 54 | LY | *Mallotus japonicus* | 0.53 | 135 | YY | *Rhus chinensis* | 4.24 |
| 55 | LY | *Quercus serrata* | 0.38 | 136 | YY | *Sassafras tzumu* | 0.33 |
| 56 | ND | *Phyllostachys edulis* | 0.64 | 137 | YY | *Schima superba* | 11.97 |
| 57 | ND | *Loropetalum chinense* | 3.08 | 138 | YY | *Styrax confusus* | 2.21 |
| 58 | ND | *Quercus fabri* | 3.66 | 139 | YY | *Symplocos anomala* | 4.88 |
| 59 | PA | *Phyllostachys edulis* | 1.24 | 140 | YY | *Symplocos caudata* | 1.19 |
| 60 | PA | *Dalbergia hupeana* | 3.80 | 141 | YY | *Symplocos heishanensis* | 5.97 |
| 61 | PA | *Hepatcodium miconioides* | 1.53 | 142 | YY | *Symplocos setchuensis* | 6.56 |
| 62 | PA | *Loropetalum chinense* | 3.76 | 143 | YY | *Symplocos stellaris* | 3.13 |
| 63 | PA | *Rhus chinensis* | 1.16 | 144 | YY | *Symplocos sumuntia* | 8.10 |
| 64 | PA | *Styrax japonicus* | 0.49 | 145 | YY | *Syzygium buxifolium* | 4.31 |
| 65 | PA | *Vitex negundo* | 3.83 | 146 | YY | *Tarenna mollissima* | 12.80 |
| 66 | PH | *Baeckea frutescens* | 3.21 | 147 | YY | *Vaccinium bracteatum* | 20.18 |
| 67 | PH | *Phyllostachys edulis* | 1.02 | 148 | YY | *Vaccinium mandarinorum* | 2.83 |
| 68 | PH | *Melastoma candidum* | 4.27 | 149 | ZG | *Phyllostachys edulis* | 0.84 |
| 69 | PH | *Rhodomyrtus tomentosa* | 1.61 | 150 | ZG | *Coriaria nepalensis* | 1.85 |
| 70 | RH | *Phyllostachys edulis* | 1.72 | 151 | ZG | *Lindera fragrans* | 4.35 |
| 71 | RH | *Loropetalum chinense* | 6.67 | 152 | ZG | *Myrsine africana* | 2.78 |
| 72 | TJ | *Phyllostachys edulis* | 1.72 | 153 | ZG | *Quercus variabilis* | 7.13 |
| 73 | TJ | *Quercus fabri* | 2.69 | 154 | ZJJ | *Phyllostachys edulis* | 2.21 |
| 74 | XF | *Baeckea frutescens* | 2.35 | 155 | ZJJ | *Cyclobalanopsis multinervis* | 1.95 |
| 75 | XF | *Phyllostachys edulis* | 0.46 | 156 | ZY | *Phyllostachys edulis* | 1.28 |
| 76 | XF | *Camellia sinensis* | 0.05 | 157 | ZY | *Castanopsis sclerophylla* | 1.69 |
| 77 | XF | *Rhodomyrtus tomentosa* | 0.78 | 158 | ZY | *Cunninghamia lanceolata* | 9.42 |
| 78 | XN | *Phyllostachys edulis* | 2.31 | 159 | ZY | *Cyclobalanopsis glauca* | 20.87 |
| 79 | XN | *Celtis julianae* | 13.40 | 160 | ZY | *Loropetalum chinense* | 3.86 |
| 80 | XN | *Quercus fabri* | 1.89 | 161 | ZY | *Pinus massoniana* | 3.21 |
| 81 | XW | *Phyllostachys edulis* | 1.11 | 162 | ZY | *Rhododendron simsii* | 0.23 |
| 82 | YA | *Phyllostachys edulis* | 0.60 | 163 | ZY | *Rhodomyrtus tomentosa* | 1.73 |

**Table S4** Subtropical tree species that exhibiting significant N, P, or N:P homeostasis in the leaves.

| **Species** | **Slope (estimate)** | **std.error** | **statistic** | ***P* value** | **Homeostasis** | **Functional Group** |
| --- | --- | --- | --- | --- | --- | --- |
| *Artemisia ordosica* | -0.22298 | 0.100875 | -2.21046 | 0.04026 | N | DeciBr |
| *Pinus halepensis* | -0.14117 | 0.054779 | -2.57706 | 0.013093 | N | Conifer |
| *Quercus suber* | -0.13328 | 0.058125 | -2.29293 | 0.02836 | N | EverBr |
| *Rhododendron racemosum* | -0.10835 | 0.047962 | -2.25912 | 0.047439 | N | EverBr |
| *Quercus ilex* | -0.08736 | 0.034566 | -2.52742 | 0.013596 | N | EverBr |
| *Rubus idaeus* | 0.135338 | 0.039479 | 3.428083 | 0.026582 | N | DeciBr |
| *Fagus sylvatica* | 0.207428 | 0.050042 | 4.145063 | 0.000501 | N | DeciBr |
| *Pinus massoniana* | 0.226648 | 0.08215 | 2.758973 | 0.012105 | N | Conifer |
| *Pinus pinea* | 0.184896 | 0.07179 | 2.57552 | 0.01691 | P | Conifer |
| *Picea abies* | 0.219717 | 0.072897 | 3.014074 | 0.01178 | P | Conifer |
| *Quercus fabri* | 0.238751 | 0.100293 | 2.380525 | 0.020184 | P | DeciBr |
| *Artemisia ordosica* | -0.22298 | 0.100875 | -2.21046 | 0.04026 | NP | DeciBr |
| *Pinus halepensis* | -0.14117 | 0.054779 | -2.57706 | 0.013093 | NP | Conifer |
| *Quercus suber* | -0.12939 | 0.060153 | -2.15099 | 0.039132 | NP | EverBr |
| *Rhododendron racemosum* | -0.10835 | 0.047962 | -2.25912 | 0.047439 | NP | EverBr |
| *Quercus ilex* | -0.09794 | 0.035288 | -2.77529 | 0.007312 | NP | EverBr |
| *Rubus idaeus* | 0.135338 | 0.039479 | 3.428083 | 0.026582 | NP | DeciBr |
| *Pinus massoniana* | 0.226648 | 0.08215 | 2.758973 | 0.012105 | NP | Conifer |

Note: the source data is from Tian et al.^[3]^

**Table S5** Site description for large-scale investigations in this study

| Site | Abbreviation | Elevation (m) | Mean annual temperature  (MAT, ℃) | Mean annual precipitation  (MAP, mm) |
| --- | --- | --- | --- | --- |
| Chishui, Guizhou | CS | 467.3 | 18.1 | 1195.7 |
| Guangshan, Henan | GS | 180.8 | 15.4 | 1027.5 |
| Huangshan, Anhui | HS | 134.5 | 16.4 | 1670 |
| Heishidu, Anhui | HSD | 130.8 | 15.3 | 1300 |
| Huitong, Hunan | HT | 331.2 | 16.8 | 1268 |
| Hezhou, Guangxi | HZ | 624.5 | 19.9 | 1558.1 |
| Jiaoling, Guangdong | JL | 394.9 | 22.3 | 1946.1 |
| Jiangshan, Zhejiang | JS | 147.4 | 16.8 | 1850.9 |
| Linan,Zhejiang | LA | 109 | 16.4 | 1628.6 |
| Laifeng, Hubei | LF | 659.3 | 15.8 | 1400 |
| Liping, Guizhou | LP | 726.2 | 16.0 | 1325.9 |
| Lishui, Zhejiang | LS | 672.4 | 17.9 | 1405.8 |
| Liyang, Jiangsu | LY | 127.1 | 15.4 | 1149.7 |
| Panan, Zhejiang | PA | 895.6 | 15.7 | 1468.8 |
| Pinghe, Fujian | PH | 536.8 | 19.4 | 1800 |
| Renhua, Guangdong | RH | 166 | 19.6 | 1665 |
| Taojiang, Hunan | TJ | 84.8 | 16.8 | 1648 |
| Wuyishan, Fujian | WYS | 253.9 | 12.5 | 2000 |
| Xinfeng, Jiangxi | XF | 311.8 | 21.5 | 1550 |
| Xianning, Hubei | XN | 155.1 | 16.8 | 1577.4 |
| Xingwen, Sichuan | XW | 673.9 | 18 | 1432.6 |
| Yongan, Fujian | YA | 225.1 | 17 | 1762 |
| Yifeng, Jiangxi | YF | 83.8 | 17.2 | 1750 |
| Yuyao, Zhejiang | YY | 136.6 | 16.2 | 1547 |
| Zigui, Hubei | ZG | 453.3 | 16.8 | 1086.6 |
| Zhangjiajie, Hunan | ZJJ | 572.3 | 17 | 1400 |
| Ziyuan, Guangxi | ZY | 538.6 | 16.7 | 1736 |

**Table S6 Primers for reverse transcription quantitative PCR (qPCR) in this study.**

| **Gene** | **Gene ID** | **Name** | **F** | **R** |
| --- | --- | --- | --- | --- |
| *PePHT1-4* | PH02Gene21291 | *qPePHT1-4* | GCTAGGAGCCTAGCCATCAAA | GAGTACTAACCCAGCAAGAAAGAGA |
| *PePHT1-6* | PH02Gene37931 | *qPePHT1-6* | TCCCCTCCTCCTTGCTTATAA | AGATGGATGGATGGCTGGCT |
| *PePHT1-8* | PH02Gene44007 | *qPePHT1-8* | TTAATATTCGTCAAGGATTCCTCAG | CGTACATGCACTAGACAGCGC |
| *PePHT1-9* | PH02Gene44008 | *qPePHT1-9* | GGTTCGTCTGCACGTTCCTC | CGCCATCTGAGACTCCGACA |
| *PeNPF2.4* | PH02Gene11942 | *qPeNPF2.4* | AGCTTATGATCCTGGGCGTCT | GTGCTCTGGGATCTCCTTGTAG |
| *PeNPF5.18* | PH02Gene38893 | *qPeNPF5.18* | ACTACTACTACGCCTTCCTCACCG | CAATTTGCATCATGCCTACCC |
| *PeNPF6.4* | PH02Gene21967 | *qPeNPF6.4* | TGGCTGGACAGGTTCTACTGG | CCGACGTGCTGGGCTTG |
| *PeNPF7.14* | PH02Gene45278 | *qPeNPF7.14* | ACCTGGACTACTACTTTTGGCTCT | ATTTGCTTGGGCGTGTATCG |
| Internal reference primers | | qPeGAPDH | CAAGGCTGTTGGCAAGGTTC | CATATGAGGCAGACTTCTCGATTC |

**References:**

[1] K. Asada, T. Kanda, N. Yamashita, M. Asano, S. Eguchi, Interpreting stoichiometric homeostasis and flexibility of soil microbial biomass carbon, nitrogen, and phosphorus. *Ecological Modelling* **2022**, *470*, 110018.

[2] Z. Tang, W. Xu, G. Zhou, Y. Bai, J. Li, X. Tang, D. Chen, Q. Liu, W. Ma, G. Xiong, H. He, N. He, Y. Guo, Q. Guo, J. Zhu, W. Han, H. Hu, J. Fang, Z. Xie, Patterns of plant carbon, nitrogen, and phosphorus concentration in relation to productivity in China’s terrestrial ecosystems. *Proceedings of the National Academy of Sciences* **2018**, *115*, 4033.

[3] D. Tian, Z. Yan, B. Schmid, J. Kattge, J. Fang, B. D. Stocker, Environmental versus phylogenetic controls on leaf nitrogen and phosphorous concentrations in vascular plants. *Nature Communications* **2024**, *15*, 5346.

[4] H. Gong, W. Song, J. Wang, X. Wang, Y. Ji, X. Zhang, J. Gao, Climate factors affect forest biomass allocation by altering soil nutrient availability and leaf traits. *Journal of Integrative Plant Biology* **2023**, *65*, 2292.
